# The Two *Caenorhabditis elegans* UDP-Glucose:Glycoprotein Glucosyltransferase Homologues Have Distinct Biological Functions

**DOI:** 10.1371/journal.pone.0027025

**Published:** 2011-11-02

**Authors:** Lucila I. Buzzi, Sergio H. Simonetta, Armando J. Parodi, Olga A. Castro

**Affiliations:** 1 Laboratory of Glycobiology, Fundación Instituto Leloir, Buenos Aires, Argentina; 2 Laboratory of Genetics and Molecular Physiology, Fundación Instituto Leloir, Buenos Aires, Argentina; 3 Instituto de Investigaciones Bioquímicas de Buenos Aires, Consejo Nacional de Investigaciones Científicas y Técnicas, Buenos Aires, Argentina; 4 School of Sciences, University of Buenos Aires, Buenos Aires, Argentina; Iowa State University, United States of America

## Abstract

The UDP-Glc:glycoprotein glucosyltransferase (UGGT) is the sensor of glycoprotein conformations in the glycoprotein folding quality control as it exclusively glucosylates glycoproteins not displaying their native conformations. Monoglucosylated glycoproteins thus formed may interact with the lectin-chaperones calnexin (CNX) and calreticulin (CRT). This interaction prevents premature exit of folding intermediates to the Golgi and enhances folding efficiency. Bioinformatic analysis showed that in *C. elegans* there are two open reading frames (F48E3.3 and F26H9.8 to be referred as *uggt-1* and *uggt-2*, respectively) coding for UGGT homologues. Expression of both genes in *Schizosaccharomyces pombe* mutants devoid of UGGT activity showed that *uggt-1* codes for an active UGGT protein (CeUGGT-1). On the other hand, *uggt-2* coded for a protein (CeUGGT-2) apparently not displaying a canonical UGGT activity. This protein was essential for viability, although cnx/crt null worms were viable. We constructed transgenic worms carrying the *uggt-1* promoter linked to the green fluorescent protein (GFP) coding sequence and found that CeUGGT-1 is expressed in cells of the nervous system. *uggt-1* is upregulated under ER stress through the *ire-1* arm of the unfolded protein response (UPR). Real-time PCR analysis showed that both *uggt-1* and *uggt-2* genes are expressed during the entire *C. elegans* life cycle. RNAi-mediated depletion of CeUGGT-1 but not of CeUGGT-2 resulted in a reduced lifespan and that of CeUGGT-1 and CeUGGT-2 in a developmental delay. We found that both CeUGGT1 and CeUGGT2 play a protective role under ER stress conditions, since 10 µg/ml tunicamycin arrested development at the L2/L3 stage of both *uggt-1(RNAi)* and *uggt-2(RNAi)* but not of control worms. Furthermore, we found that the role of CeUGGT-2 but not CeUGGT-1 is significant in relieving low ER stress levels in the absence of the *ire-1* unfolding protein response signaling pathway. Our results indicate that both *C. elegans* UGGT homologues have distinct biological functions.

## Introduction

The endoplasmic reticulum (ER) is the subcellular compartment where glycoproteins acquire their tertiary and quaternary structures. The quality control of glycoprotein folding allows cells to discriminate between native and non native protein conformations, selectively transporting properly folded proteins to their final destinations through the secretory pathway, or alternatively, retrotranslocating proteins recognized by cells as irreparably misfolded or incompletely formed glycoprotein complexes to the cytosol to be degraded by proteasomes. The *N*-glycosylation of proteins starts with the “en bloc” addition of a glycan of composition Glc_3_Man_9_GlcNAc_2_ to polypeptide chains in the ER lumen by the oligosaccharyltransferase complex. The glycan is first processed by two ER-resident enzymes, glucosidase I which removes the outermost glucose residue and glucosidase II (GII) that removes the middle and innermost glucose units. An ER mannosidase (s) may also excise several mannose units. Monoglucosylated glycoproteins bearing glycans of compositions Glc_1_Man_7–9_GlcNAc_2_ may interact with two ER-resident lectin chaperones, membrane bound calnexin (CNX) or its soluble homologue calreticulin (CRT). Monoglucosylated *N*-glycans may also be formed by reglucosylation of deglucosylated glycans of structure Man_7–9_GlcNAc_2_ by the UDP-Glc:glycoproteinglucosyltransferase (UGGT). This enzyme is the key component of the folding quality control mechanism. It behaves as a sensor of glycoprotein conformations as it exclusively glucosylates glycoproteins not displaying their native conformations. Lectin–glycoprotein binding and unbinding as a result of the opposing activities of UGGT and GII continues until glycoproteins either acquire their native structures or, alternatively are recognized by cells as irreparably misfolded species or as complexes unable to acquire their full subunit complement. The interaction of folding intermediates, incomplete complexes and irreparably misfolded glycoproteins with the lectin-chaperones not only prevents Golgi exit of the former but also decreases the folding rate and increases folding efficiency by preventing aggregation and facilitating correct disulfide bond formation through their interaction with ERp57, a protein disulfide isomerase loosely associated with CNX and CRT [1]–[4].

The UGGTs from different organisms are rather large (about 160 kDa) monomeric soluble proteins localized to the ER. Most of them display a KDEL-like ER retention/retrieval signal at their C-terminus. UGGT specifically utilizes UDP-Glc as sugar donor and needs millimolar Ca^2+^ concentrations for activity [1]. Bioinformatics analysis and biochemical studies of mammalian, insect and yeast UGGTs suggested that it is composed of at least two domains [2]. The N-terminal domain comprises 80% of the molecule, has no homology to other known proteins and is presumably involved in non-native conformer recognition. The C-terminal domain displays a similar size and significant homology to members of glycosyltransferase family 8. UGGT C-terminal domains from different species share a significant similarity (65–70%), but no such similarity occurs between the N-terminal ones. For instance, *Rattus norvegicus* and *Drosophila melanogaster* UGGT N-terminal domains share a 32.6% similarity but they only show a respective 15.5 and 16.3% similarity with the same portion of *S. pombe* UGGT. Although there are both structural and experimental evidence supporting the idea that the C-terminal domain is the catalytic portion of the enzyme, the often advanced notion that the N-terminal domain is responsible for recognition of nonnative conformers has not been firmly established yet [3].

The genome of *Caenorhabditis elegans* codes for proteins homologous to all participants in the quality control of glycoprotein folding mentioned above, although not in all cases their role in that mechanism has been confirmed. This role characterization is necessarily required in the case of UGGT, as several unicellular and multicellular organisms express UGGT-like proteins lacking enzymatic activity and of unknown function. This is the case in *Saccharomyces cerevisiae* in which the single protein encoded in its genome with UGGT homology (Kre5p), lacks enzymatic activity [4]. On the other hand, whereas in *Schizosaccharomyces pombe, Drosophila melanogaster, Trypanosoma cruzi* and plants an enzymatically active UGGT is encoded by a single gene [5], [6], [7], there are two homologues coding for UGGT-like proteins in Euteleostomi, which is a successful clade that includes more than 90% of the living species of vertebrates [8], and at least in some species of nematodes belonging to the genus Caenorhabditis. Bioinformatics analysis showed that in *C. elegans* there are two open reading frames (F48E3.3 and F26H9.8 hereinafter referred to as *C. elegans uggt-1* and *uggt-2* genes respectively) coding for UGGT homologues (CeUGGT-1 and CeUGGT-2). Both proteins share a 40% identity (52% and 31% in the C-terminal and N-terminal domains, respectively). It is still unknown if both genes codes for active UGGTs or if only one of them displays UGGT activity.

There are few reports, that will be further discussed below, showing that UGGT expression is crucial for mammalian embryonic development but not for single cell viability. As most studies on the role of UGGT in the glycoprotein folding quality control and its relevance in cell survival under normal and stress conditions were performed in either mammalian or yeast single cells we decided to further characterize the significance of the enzyme in a simple multicellular organism as *C. elegans.* Here we report a characterization of the *C. elegans* protein coded by the open reading frame (ORF) F48E3.3, its enzymatic activity and its body pattern expression. We also analyzed the expression of both ORF F48E3.3 and F26H9.8 under normal and stress conditions, the phenotypes associated with the loss of function of the proteins encoded by ORFs F48E3.3 and F26H9.8 in gene silencing experiments and the characterization of the homozygous ORF F26H9.8 partial deletion mutant. We conclude that both proteins have different biological functions.

## Materials and Methods

### Media strains and reagents

The following *C. elegans* strains were used in this study: Bristol strain N2 as standard wild-type strain [9], KP3948 (*eri-1*(mg366) IV; *lin15B* (n744) X); SJ30 [*ire-1*(zcI4) II; zcIs4 V], RB772 [*atf-6* (ok551) X], RB545 [*pek-1* (ok275) X] kindly provided by the *C. elegans* Genetics Center (CGC; Minneapolis, MN) and VC1961 [*uggt-2 (ok2510)/*hT2[bli-4(e937) let-?(q782) qIs48] kindly provided by *C.elegans* Reverse Genetics Core Facility at the University of British Columbia. N2 and mutants were grown at 20°C in nematode growth medium (NGM) plates seeded with OP50 unless otherwise indicated. *gpt1/alg6 S. pombe* (Sp61G4A (h-, *ade6-M210*, *ade1*, *leu1-32*, *ura4-*D18, *gpt1::ura4*-D1684, *alg6::ura4+*) was used for heterologous expression [10]. *S. pombe* cells were grown at 28°C in YEA medium or MM medium supplemented with adenine or leucine as needed [11]. *Escherichia coli* strain STBL3 (Invitrogen, Carlsbad, CA) was grown in LB medium with 100 µg/ml ampicillin when needed. Reagents for yeast media were obtained from Difco Laboratories (Detroit, MI). [^14^C]Glc (301 Ci/mol) was from Perkin Elmer Life and Analytical Sciences (Boston, MA). N-Methyl-1-deoxynojirimycin (NMDNJ) was from Research Chemicals (North York, ON, Canada). Enzymes used for DNA procedures were from New England Biolabs (Ipswich, MA). Unless otherwise stated, all other reagents were from Sigma (St. Louis, MO). UDP-[^14^C]Glc was synthesized as previously reported with slight modifications [12].

### Cloning and expression of *uggt-1* and *uggt-2* in *gpt1/alg6 S. pombe* cells

The open reading frames F48E3.3 (Chromosome X: 7500939−7495639) and F26H9.8 (CHROMOSOME I: 9317728–9327204) present in *C. elegans* ORFeome Database code for UGGT homologues. Codon-optimized versions of *uggt-1* and *uggt-2* for expression in yeast were synthesized by GenScript (Piscataway, NJ). The sequences of *uggt-1* and *ugg-t-2* were optimized and the codon usage bias was increased by upgrading the Codon Adaptation Index (CAI) from 0.66 to 0.92 for *uggt-1* and from 0.70 to 0.92 for *uggt-2*. The Frequency of Optimal Codons was increased from 48 to 86% for *uggt-1* and from 56 to 87% for *uggt-2*. The GC content and unfavorable peaks have been optimized to prolong the half-lives of mRNAs. The Stem-Loop structures, which impact ribosomal binding and stability of mRNA, were broken. These optimized sequences were cloned in *S. pombe nmt* promoter-driven expression vector pREP3x (LEU2), kindly provided by Dr. Susan Forsburg (Department of Biological Sciences, University of Southern California, Los Angeles, CA). A CAI of 1 is considered perfect in the desired organism and a CAI >0.9 is considered as very good. The CAI value obtained for both genes was 0.92, therefore the expected level of expression for both genes was the same and very good. The plasmids were electroporated into *gpt1/alg6 S. pombe* cells and transformants were selected on MM plates plus adenine containing 15 µM thiamine.

### Expression of GFP fusion constructs

*uggt-1* and *uggt-2* promoter sequences were PCR amplified using pDONR P4-P1R vectors containing ORF F48E3.3 and ORF F26H9.8 promoters (Chromosome X: 7501192−7500938) and (CHROMOSOME_I 9317727−9314480), obtained from the *C. elegans* Promoter Library from Open Biosystems (Alabama USA), with a high fidelity polymerase (Hot start KOD, Roche) and cloned into the pPD95.75 polylinker (Addgene) immediately upstream of the GFP coding region to generate the Promoter *uggt-1::gfp* and Promoter *uggt-2::gfp* fusion constructs. The constructions were microinjected at 50–75 ng/µl concentrations into the syncytial gonad of several young wild-type adult hermaphrodites together with 100 ng/µl of the plasmid pRF4 containing the dominant marker rol-6 (su1006) [13]. Several transgenic animals expressing both GFP and *rol-6* marker were obtained and grown independently. Worms in stable transgenic lines were visualized by fluorescence confocal microscopy using an LSM510 Meta confocal microscope (Carl Zeiss, Oberkochen, Germany). Images were acquired with LSM software (Carl Zeiss) using a 20 x plan apochromat objective.

## Methods

Strong acid hydrolysis was performed as described before [14]. Whatman 1 paper was used for chromatography. Solvents used were: (A) 1-propanol/nitromethane/water (5∶2∶4), (B) 1-butanol/pyridine/water (10∶3∶3). UGGT was assayed using microsomes as enzyme source and 8 M urea-denatured thyroglobulin as acceptor, as described previously [15]. *S. pombe* and rat liver microsomes were prepared as already described [1], [14]. Protein concentrations were determined by Bio-Rad Protein Assay as described by the manufacturer.

### Preparation of *C. elegans* membrane fraction

Packed mixed-stage worms, grown in liquid culture (S basal medium) using concentrated OP50 bacteria as food, were resuspended in equal volume of membrane buffer (0.25 M sucrose, 5 mM EDTA, 20 mM imidazole-HCl buffer, pH 7.5, supplemented with protease inhibitors (1 mM phenylmethylsulfonylfluoride, 1 µM pepstatin, 10 µM leupeptin, 1 mM tosylphenylalanylchloromethylketone, 1 µM *N* [1-[*N*-[(L-3-trans-carboxyoxirane-2-carbonyl)-L-leucyl]amino]-4-guanidinobutane]). Cells were disrupted by vortexing them several times (1 min each) with 0.5-mm glass beads. The suspension was sonicated twice, at 0.4 volts output for 20 sec. The homogenate was centrifuged at 1500 x *g* for 10 min at 4°C, and the resulting supernatant was centrifuged at 100,000 x *g* for 60 min at 4°C. The pellet was suspended in membrane buffer supplemented with protease inhibitors and stored in aliquots at −70°C.

### In vivo labeling of *S. pombe* cells with [^14^C]Glc

Pulse labeling of *S. pombe gpt1*/alg6 cells transformed with pREP3X-*uggt-1*, pREP3X-*uggt-2* and pREP3X*gpt1^+^* with [^14^C]Glc was performed as described before [14]. Cells were preincubated for 60 min in 2.5 mM NMDNJ at 24°C, the final 5 min in presence of 5 mM dithiothreitol (DTT), and pulsed for 30 min in 5 mM Glc containing 150 µCi of [^14^C]Glc. Isolation of endo-β-N-acetylglucosaminidase H (Endo H)-sensitive glycans was performed as already described [14].

### UGGT assay

Except where otherwise stated, the incubation mixtures contained, in a total volume of 50 µl**,** 0.2 mg of 8 M urea-denatured bovine thyroglobulin, 10 mM CaCl_2_, 3 µCi UDP-[^14^C]Glc, 0.4% Lubrol, 1 mM NMDNJ, and 150–200 µg of proteins of the samples being assayed. Reactions were stopped by the addition of 1 ml of 10% trichloroacetic acid, and the mixtures were further processed as described before [15].

### Real-time analysis

Worms grown as indicated in each experiment were harvested by washing them from the plates and further washed twice with M9 medium. Total RNA was prepared using Qiagen RNeasy extraction kit. For real-time RT–PCR, equal amounts of RNA (5 µg) were added to 1X reaction buffer, random primers, and SuperScript II reverse transcriptase (Invitrogen) to generate a cDNA template for PCR following manufacturer's protocols. The resulting cDNA was used for real-time PCR (Stratagene MX300 sp), using the hot start Platinum Taq DNA polymerase (Invitrogen) and SYBRGreen and ROX (Invitrogen) as fluorescent dyes. Three independent biological samples were analyzed in each experiment. Primers used for real time PCR are as follows for *uggt-1*, uggt-1F 5′GATTCAACGTCACTTTTAGCCG3′ and uggt-1R 5′TTTTCCCAATTCAATGTACCGAC3′; for *uggt-2*, uggt-2F 5′CAACATGCTTCACGAAGTTCC3′ and uggt-2R 5′GCCGAGTTCAGTTTTGGTTC3′; for *hsp-4*, hsp-4F 5′GCAGATGATCAAGCCCAAAAAG3′ and hsp4R 5′GCGATTTGAGTTTTCATCTGATAGG3′; for *ama-1*, ama-1F 5′CCTACGATGTATCGAGGCAAA3′ and ama-1R 5′CCTCCCTCCGGTGTAATAATG3′.

### Yeast RNA preparation and RT-PCR

*S. pombe gpt1/alg6* cells carrying pREP3X-*uggt-1,* pREP3X-*uggt-2* or pREP3X-*gpt1+* were grown in 5 ml MMA to mid-log phase and total RNA was prepared using Qiagen RNeasy extraction kit. RNA samples were treated with RQ1 RNase free DNase (Promega) in 5 mM MgCl_2_ and 50 mM Tris-HCl, pH 8.0, at 37°C for 30 min followed by heat inactivation (15 min at 75°C). Equal amounts of RNA (1 µg) were added to 1X reaction buffer, random primers, dNTPs and SuperScript II reverse transcriptase (Invitrogen) to generate a cDNA template for PCR according to the manufacturer's protocol. The resulting cDNA was used as template for a PCR reaction (4 min at 94°C, 30 cycles of 45 sec at 94°C, 45 sec at 53°C, 1 min at 72°C and 10 min at 72°C) using the hot start Platinum Taq DNA polymerase (Invitrogen). Primers used for RT–PCR were as follows for *uggt-1OPT*, uggt-1FWDRT 5′GATGAGAAGACAACTTATGC3′ and uggt-1REVRT 5′TATCATAATCAGAGTTGA3′ for *uggt-2OPT*, uggt-2FWDRT 5′GTAGTGTCGAATGGATTG3′ and uggt-2REVRT 5′TGTCATAGTTCTTGCCAC3′; for *gpt1,* gpt1FWD 5′AGGAATTGATGGATATGGATT3′ *and* gpt1REV 5′AAATCGATTGTTTTGGCG3′ for *act-1,* act1FWD 5′GGCTCTGGTATGTGCAAAG3′ and act-1 REV 5′CAACGTTACCGTACAAATC3′.

### Expression of *uggt-1* and *uggt-2* during development

Synchronized L1 stage wild type N2 worms were seeded onto NGM plates and allowed to develop to adults at 20°C. Three plates containing 10000 worms each were processed after 24, 48 and 72 h to prepare RNA samples representing L2/L3, L4 and adult stages. About 24000 L1 stage wild type N2 worms were immediately processed to prepare RNA samples representing L1 stage. *uggt-1* and *uggt-2* expression levels were determined by real-time PCR. Three independent samples were analyzed in each experiment.

### RNA interference

An L1 synchronized population of KP3948 worms was seeded onto RNAi plates and grown to adult hermaphrodites (P0). P0 gravid hermaphrodites were used to obtain an L1 synchronized population (F1) that was again seeded onto RNAi plates. F1 Gravid hermaphrodites were synchronized to obtain an L1 synchronized population (F2). F2 worms were used for lifespan analysis. RNAi experiments were performed as described before [16]. ORF-RNAi clones were obtained cloning the entire open reading frame of each gene in the feeding vector L4440 and transforming them in the feeding bacteria HT115(DE3). Three independent biological samples were analyzed in each experiment.

### Expression of *uggt-1* and *uggt-2* under tunicamycin (TN) stress

Synchronized L1 populations of wild type N2 worms were seeded onto NGM plates (containing about 6000 worms each) and allowed to develop to L2/L3 stage or to adults at 20°C. Worms from each plate were washed twice with M9 medium and resuspended in 5 ml of M9 containing or not 5 µg/ml TN and treated for 6 h at 20°C. The level of expression of *uggt-1*, *uggt-2* and *hsp-4* was determined by real-time PCR.

### TN sensitivity test

Synchronized embryos of the KP3948 strain were hatched overnight in M9 medium to obtain an L1 synchronized population that was seeded onto RNAi plates and grown to adult hermaphrodites (P0). P0 gravid hermaphrodites were used to obtain an L1 synchronized population (F1) that was seeded onto RNAi plates and allowed to grow to adults. Gravid hermaphrodites were transferred to RNAi plates containing several TN concentrations (0, 2.5, 5 or 10 µg/ml) for 6 h to lay eggs after which adults were removed. The developmental stages of F2 were analyzed for 5 days at intervals of 24 h and the percentages of L1, L2/L3, L4 and adults were determined.

### Characterization of VC1961 strain

N2 wild type and VC1961 *uggt-2* (ok2510)/*hT2* worms were synchronized, seeded onto NGM plates and allowed to grow to adult hermaphrodites. Gravid hermaphrodites were transferred to NGM plates for 6 h to lay eggs after which adults were removed. The developmental stages of the F1 were analyzed for 5 days at intervals of 24 h and the percentages of eggs, L1, L2/L3, L4 adults and gravid hermaphrodites were determined.

## Results

### *C. elegans* expresses an active UGGT

**Figure 1:**
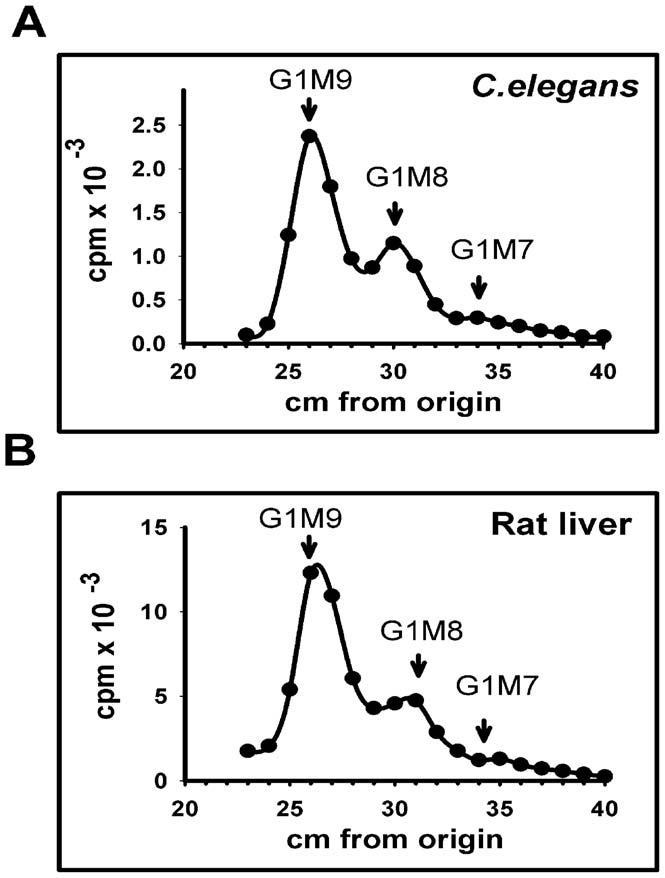
UGGT activity in *C. elegans*. *C. elegans* (A) and rat liver (B) microsomal proteins were incubated in a mixture that contained 5 mM Tris-maleate buffer, pH 7.5, 10 mM CaCI_2_, 0.6% Triton X-100, 5 mM NMDNJ and 3 µCi UDP-[^14^C]Glc, at 24°C (A) and 37°C (B) for 60 min. Glycans obtained by Endo H treatment were subjected to paper chromatography with solvent *A*. Standards G1M9: Glc_1_Man_9_GlcNAc; G1M8: Glc_1_Man_8_GlcNAc and G1M7: Glc_1_Man_7_GlcNAc.

As mentioned above, UGGT-like proteins lacking enzymatic activity are expressed in both mammalian and yeast cells. To confirm the presence of an UGGT activity in *C. elegans* we prepared microsomes of wild type N2 worms grown in liquid medium and incubated them with denatured thyroglobulin. Endo H-sensitive glycans were run on paper chromatography with appropriate standards [1]. An experiment using rat liver microsomes as enzyme source was included for comparison (Figure 1).

*C. elegans* microsomes incubated with UDP-[^14^C]Glc yielded products that migrated as Glc_1_Man_9_GlcNAc, Glc_1_Man_8_GlcNAc and (barely detectable) Glc_1_Man_7_GlcNAc standards on paper chromatography (Figure 1A). The same compounds were obtained when using rat liver microsomes (Figure 1B) thus confirming the presence of a glucosylating activity in *C. elegans* microsomes.

We also analyzed the cation requirements and optimum pH value of the worm UGGT activity: the enzyme required Ca^2+^ for activity (optimum 5–10 mM). Neither Mg^2+^ nor Mn^2+^ could replace Ca^2+^. The enzyme showed an almost neutral optimum pH value. (Figure S1).

### The ORF F48E3.3 codes for an UGGT activity

As we had confirmed that *C. elegans* displays an UGGT activity either *uggt-1* or *uggt-2* or both must code for an active enzyme. To study this point we expressed both genes in a *S. pombe* mutant lacking UGGT activity and transferring Man_9_GlcNAc_2_ to protein (*gpt1*/*alg6*). As *C. elegans* has a codon usage quite different from yeast we cloned optimized versions of *uggt-1* and *uggt-2* in the *S. pombe* expression vector pREP3X (LEU2) (see Materials and Methods).

**Figure 2:**
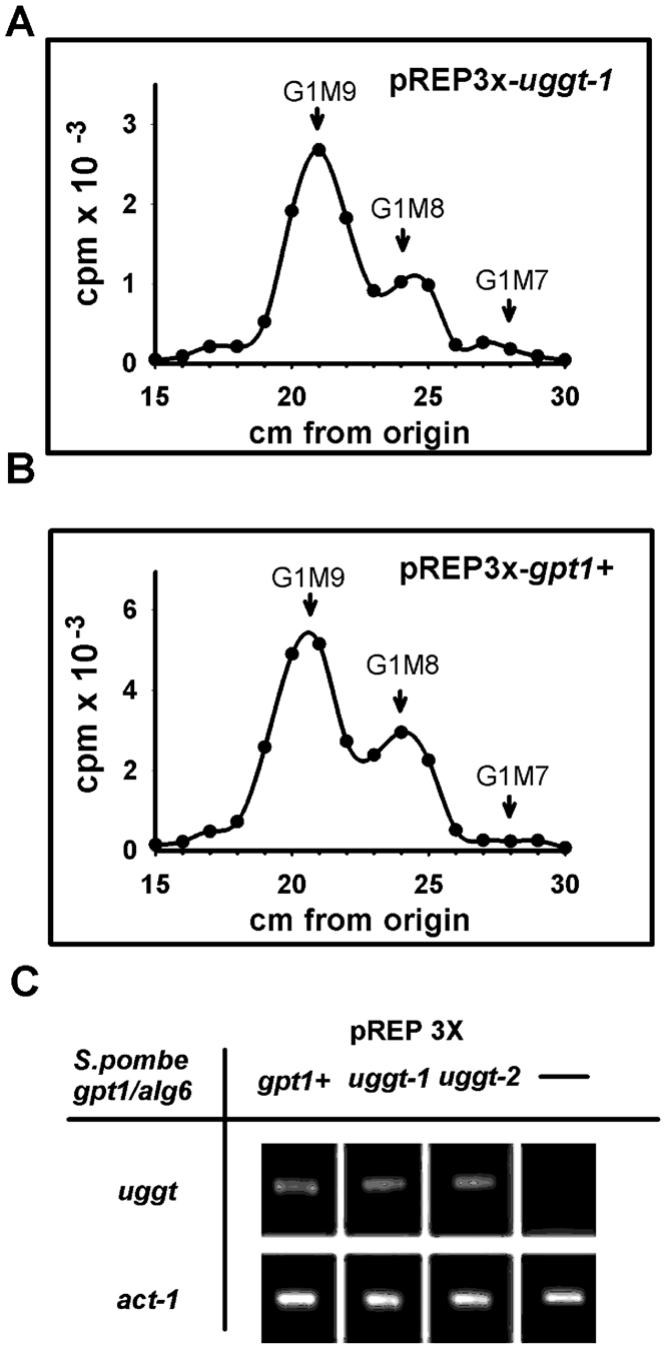
*uggt-1* codes for an active UGGT activity. In vitro assays. *S. pombe* (*gpt1*/*alg6*) microsomal proteins prepared from cells carrying pREP3X-*uggt-1* (A) or pREP3X-*gpt1* (B) were incubated in a mixture containing 5 mM Tris-maleate buffer, pH 7.5, 10 mM CaCI_2_, 0.6% Triton X-100, 5 mM NMDNJ and 3 µCi UDP-[^14^C]Glc (300 Ci/mol), at 24°C for 30 min. Glycans obtained by Endo H treatment were subjected to paper chromatography with solvent *A*. (C) Total RNA was isolated from *S. pombe gpt1/alg6* cells carrying pREP3X-*gpt1+,* pREP3X-*uggt-1,* pREP3X-*uggt-2* or pREP3X and used to generate cDNA. RT-PCR analysis was performed with specific primers for *gpt-1, uggt-1, uggt-2* and β-actin mRNAs.

Microsomes from *S. pombe gpt1/alg6* cells carrying pREP3X-*uggt-1,* pREP3X-*uggt-2,* pREP3X-*gpt1+ or* pREP3X were assayed for UGGT activity with UDP-[^14^C]Glc as sugar donor and denatured thyroglobulin as acceptor. Labeled glycans formed were analyzed as described above [1]. Microsomes derived from cells carrying pREP3X-*uggt-1* or pREP3X-*gpt1+,* yielded products that migrated as Glc_1_Man_9_GlcNAc, Glc_1_Man_8_GlcNAc and Glc_1_Man_7_GlcNAc standards on paper chromatography (Figure 2A, B). Conversely, microsomes derived from cells carrying pREP3X and pREP3X-*uggt-2* yielded no detectable endo-H sensitive [^14^C]-glycans. To check if *uggt-2* mRNA was actually expressed in *S. pombe* cells, we performed RT analysis and found that *uggt-2* mRNA levels were similar to those of *uggt-1*mRNA and *gpt1+* mRNA (Figure 2C).

**Figure 3:**
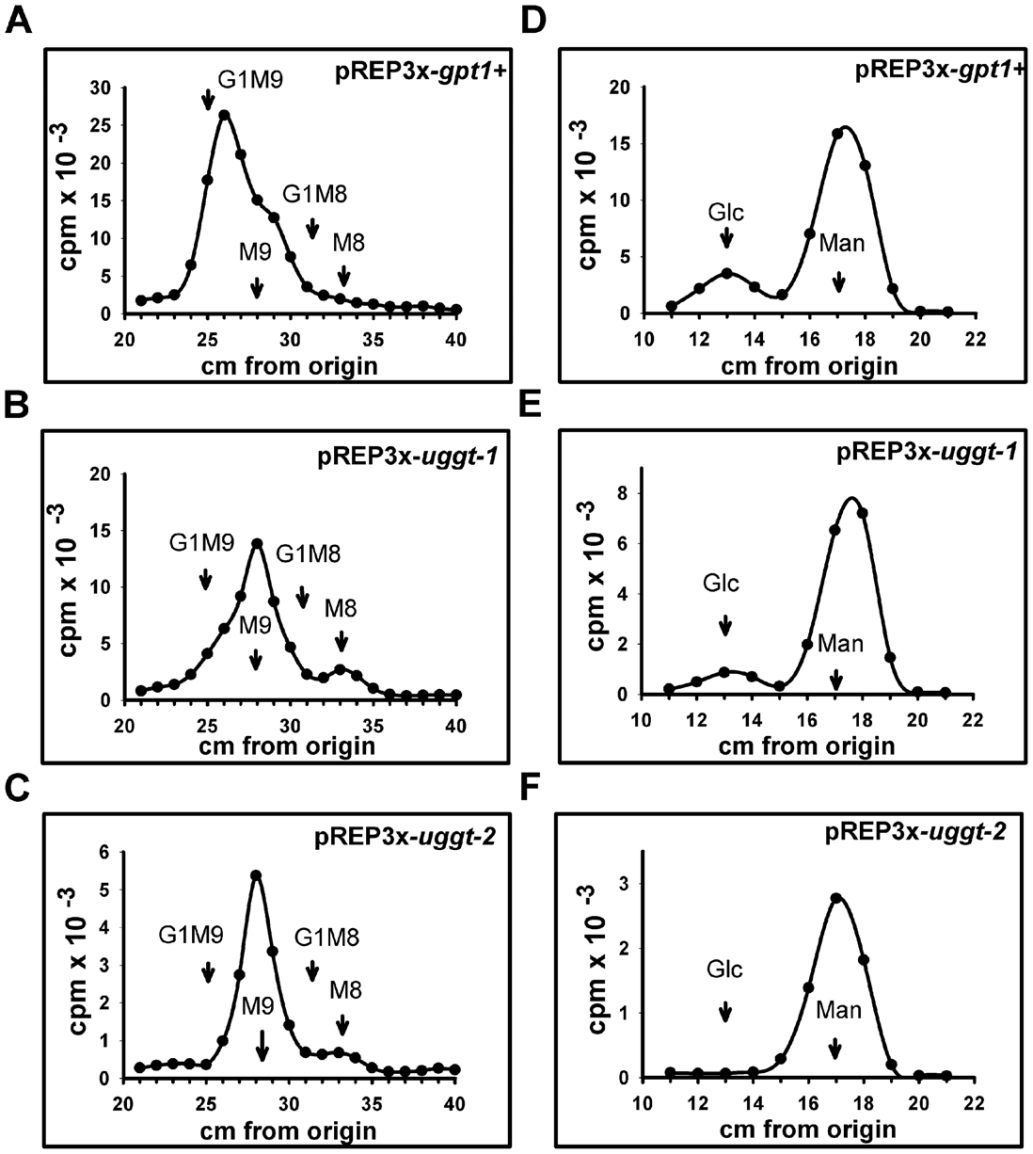
*uggt-1* codes for an active UGGT activity. In vivo assays. Indicated cells were preincubated for 60 min with NMDNJ, the final 5 min in presence of 5 mM DTT and pulsed for 30 min in 5 mM Glc with 150 µCi of [^14^C]Glc at 24°C. Glycans liberated by Endo H treatment were subjected to paper chromatography with solvent *A* (Figures 3A–C). Glycans migrating between 24 and 35 cm in panels A–C were submitted to strong acid hydrolysis and run on paper chromatography with solvent *B* (Figures 3D–F). Standards: G1M9, Glc_1_Man_9_GlcNAc; M9, Man_9_GlcNAc; G1M8, Glc_1_Man_8_GlcNAc and M8 Man_8_GlcNAc.

To further confirm the absence of activity in *alg6/gpt1 S. pombe* carrying pREP3X-*uggt-2* we labeled gpt1/alg6 *S. pombe* mutants carrying pREP3X-*gpt1+*, pREP3X-*uggt-1* and pREP3X-*uggt-2* with [^14^C]Glc and analyzed glycans synthesized *in vivo* (Figure 3). It is worth mentioning that labeling was performed in the presence of a cell permeable glucosidase II inhibitor (NMDNJ) and of DTT, a compound that prevents passage of glycoproteins synthesized from the ER to the Golgi.

*S. pombe* cells containing pREP3X-*gpt1* yielded glycans that migrated as a mixture of Glc_1_Man_9_GlcNAc, Man_9_GlcNAc, Glc_1_Man_8_GlcNAc and Man_8_GlcNAc standards (Figure 3A). The same result was obtained with cells containing pREP3X-*uggt-1* whereas those carrying pREP3X-*uggt-2* only produced compounds that migrated as Man_9_GlcNAc and Man_8_GlcNAc standards (Figure 3B, C). As the analytical system employed does not provide a neat separation between glucosylated and unglucosylated glycans we submitted substances migrating between G1M9 and G1M8 standards to strong acid hydrolysis followed by paper chromatography with solvent B. Only in the first two cases labeled glucose residues were detected.

**Figure 4:**
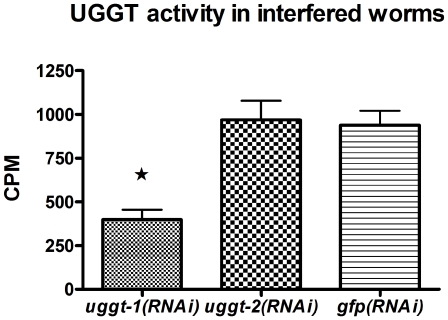
UGGT activity decreases in *uggt-1(RNAi)* worms but no decrease was observed in *uggt-2 (RNAi)* worms. *F2 uggt-1(RNAi), uggt-2(RNAi) and control gfp(RNAi)* worm microsomal proteins were incubated in a mixture that contained 5 mM Tris-HCl buffer, pH 7.5, 10 mM CaCI_2_, 0.6% Triton X-100, 5 mM NMDNJ and 3 µCi UDP-[^14^C]Glc, at 20°C for 30 min. Reactions were stopped with 1 mL of 10% of trichloroacetic acid. After centrifugation, the pellets were twice washed with 1 mL of 10% trichloroacetic acid and counted. The values shown are the mean of two independent experiments. Error bars represent standard deviations * indicates significant differences.

To check if it could be ascertained if CeUGGT-2 displayed indeed a glucosyltransferase activity we assayed microsomes derived from *uggt-1(RNAi) uggt-2(RNAi)* and *gfp(RNAi) control* worms for UGGT activity. As depicted in Figure 4 we found a 57,69±4.3% decrease in UGGT activity in *uggt-1(RNAi)* worms but no decrease in UGGT activity was found in *uggt-2(RNAi)* worms, although further work (see below) showed that *uggt-2*(*RNAi*) had effectively interfered *uggt-2* expression. This data were analyzed using one way ANOVA and Bonferroni post test analysis and showed significant differences between the level of UGGT activity in *uggt-1(RNAi)* worms and *gfp(RNAi)* control worms with a P value <0.001 and no significant differences were found between the level of UGGT activity in *uggt-2(RNAi)* and *gfp(RNAi) control* worms P>0.05. Although results presented in Figure 4 would suggest that CeUGGT-2 does not display a glucosyltransferase activity, results shown may reflect, alternatively the low level of uggt-2 expression (see below).

### *uggt-1* and *uggt-2* are expressed at different levels during development

**Figure 5:**
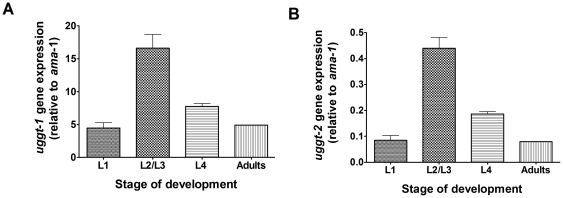
*uggt-1* and *uggt-2* expression pattern during development. Expression levels of *uggt-1* mRNA (A) and *uggt-2* mRNA (B) relative to those of *ama-1* mRNA during development as measured by Real-time PCR. The values shown are the mean of three independent experiments. Error bars represent standard deviations.

Bioinformatics analysis showed that *uggt-1* and *uggt-2* code for UGGT homologues (CeUGGT-1 and CeUGGT-2) but only CeUGGT-1 showed UGGT activity. We may envisage two possibilities, either *uggt-2* codes for a protein with an unknown function not related to UGGT activity or, alternatively, *uggt-2* is not transcribed and/or translated *in vivo*. In order to check if that both genes are actually transcribed in *C. elegans* we quantified the mRNA levels of *uggt-1* and *uggt-2* at different developmental stages using *ama-1* as reference gene. Real-time PCR analysis revealed that *uggt-1* and *uggt-2* genes are indeed transcribed throughout the *C. elegans* life cycle with a maximum at the L2/L3 stage (Figure 5). Even though *uggt-1* and *uggt-2* share the same expression pattern during development, the level of *uggt-2* transcription was much lower (at most 3%) than that of *uggt-1* at all stages.

### *uggt-1* is upregulated under ER stress conditions

**Figure 6:**
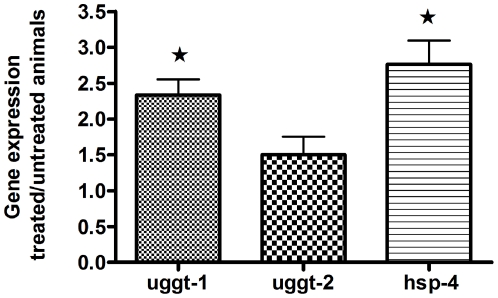
*uggt-1* but not *uggt-2* is upregulated under stress conditions. Total RNAs from untreated and 5 µg/ml TN-treated animals were isolated and the levels of *uggt-1*, *uggt-2* and *hsp4* expression were quantified by real-time PCR using *ama-1* as reference gene. Relative expression levels represents RNA expression in TN treated worms/RNA expression in untreated worms. The value obtained for untreated samples was considered as one. The values shown are the mean of three independent experiments. Error bars represent standard deviations * indicates significant differences.

An interesting possibility is that although CeUGGT-2 apparently lacks UGGT activity, it could be somehow involved in protein folding. If this were the case we would expect to detect an increase in the transcription not only of *uggt-1* but also of *uggt-2* in worms subjected to conditions triggering the accumulation of unfolded proteins in the ER. To analyze this possibility total RNA from synchronized young adult worms treated and untreated with 5 µg/ml TN for 6 h at 20°C were isolated and the expression levels of *uggt-1*, *uggt-2* and *hsp-4* (which codes for a *C. elegans* functional homologue of mammalian BiP) was quantified by Real-time PCR (Figure 6).

The analysis revealed 2.3, and 2.7 times increases in the expression of *uggt-1 and hsp-4* respectively. We analyzed these results with a 2 tails T-test for one sample and found significant differences with a P value for 0.02 for *uggt-1* and 0.03 for *hsp-4* but the lower increase found for *uggt-2* was of no statistical significance (P value of 0.18). As the maximum expression of both genes was in L2/L3 stage we also prepared total RNA from synchronized L2-L3 worms treated and untreated with 5 µg/ml TN for 6 h at 20°C as in the case of young adults worms and the expression levels of *uggt-1*, *uggt-2* and *hsp-4* was quantified by real-time PCR. The analysis revealed 2.01, and 20.64 times increases in the expression of *uggt-1 and hsp-4* respectively. We analyzed these results with a 2 tails T-test for one sample and found significant differences with a P value of 0.0034 for *uggt-1* and P<0.0001 for *hsp-4* but the minor increase found for *uggt-2* (1.13 times) was of no statistical significance (P value 0.602) (Figure S2). The high level of *hsp-4* induction has already been observed in *C.elegans* [17]. These results suggest that only CeUGGT-1 is upregulated under ER stress conditions induced by TN treatment.

### UPR activation

**Figure 7:**
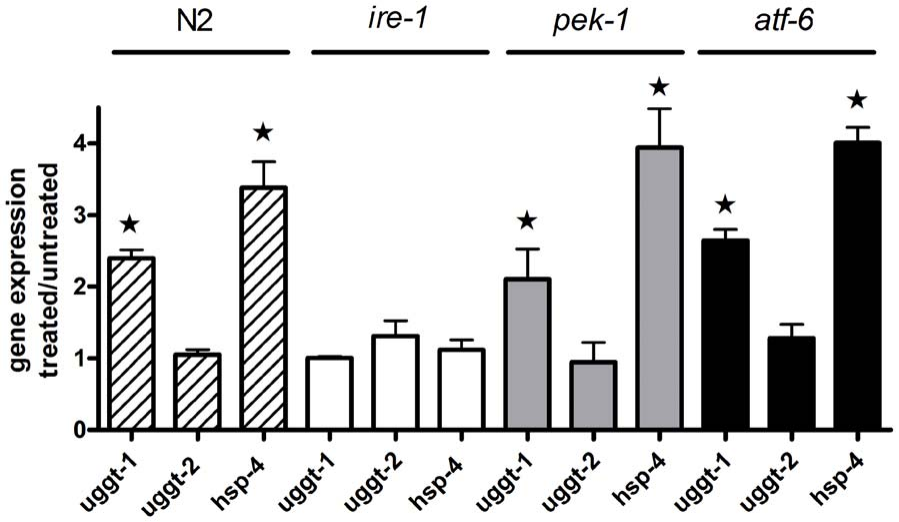
*uggt-1* expression is regulated by the *ire-1* arm of the unfolded response pathway. Total RNA from young adults, wild type, *ire-1, atf-6* and *pek-1* worms, treated and not treated with 5 µg/ml TN was extracted and the levels of mRNA coding for *uggt-1*, *uggt-2* and *hsp-4* were analyzed by real time PCR using *ama-1* as reference gene. Gene expression level represents RNA expression in TN treated worms/RNA expression in untreated worms. The value obtained for untreated samples was considered as one. The values shown are the mean of three independent experiments. Error bars represent standard deviations * indicates significant differences.

Three proteins are known to sense ER stress and activate the UPR in *C.elegans*: the ribonuclease inositol-requiring protein–1 (IRE-1), the PERK kinase homologue PEK-1, and the activating transcription factor–6 (ATF-6) [17], [18], [19], [20]. When activated by ER stress, IRE-1′s endonuclease activity is switched on, and, as a consequence, it removes an intron from xbp-1 (X-box binding protein–1) mRNA. Spliced xbp-1 encodes a transcription factor that activates expression of downstream genes, such as genes encoding chaperones and ER-associated degradation proteins [17], [18], [20], that expand the ER's folding capacity. Three cis-acting elements capable of binding to ATF-6, XBP-1 or both have been identified to date, namely ER stress-response element (ERSE-I), unfolded protein response element (UPRE) and ERSE-II. Transcription from UPRE depends solely on XBP-1 [21]. We have identified a potential UPRE element (XBP-1 binding site) GC–TGACGT-CG at positions -120 to −130 of the *uggt-1* promoter. The same cis-acting element capable of binding XBP-1 is present in the *hsp-3 and hsp-4* promoters. No sequences similar to UPRE, ERSE-I and ERSE-II have been found in the *uggt-2* promoter. The increase in *uggt-1* transcription under ER stress and the presence of a UPRE sequence close to the *uggt-1* promoter suggests that CeUGGT-1 is upregulated in response to ER stress through the ire-1 arm of the UPR. To confirm this point total RNA from young adults wild type, *ire-1, atf-6* and *pek-1* worms, treated and not treated with 5 µg/ml TN was extracted and the level of mRNAs coding for *uggt-1*, *uggt-2* and *hsp-4* was analyzed by real time PCR. Results are shown in Figure 7.

The analysis revealed 2.41, 1.04, 2.46 and 2.44 times increases in the expression of *uggt-1* in wild type, *ire-1, pek-1* and *atf-6* worms respectively. We analyzed these results with a 2 tails T-test for one sample and we found significant differences with a P value for 0.0072, 0.017 and 0.015 in wild type, *pek-1* and *atf-6* worms but the 1.04 value found in *ire-1* worms was of no statistical significance (P value of 0.12). We also analyzed the level of expression of *uggt-2* and found no significant differences between the level of expression of *uggt-2* in treated and untreated wild type, *ire-1*, *pek-1* and *atf-*6 worms (P value of 0.3050, 0.737, 0.492 and 0.078 respectively). We found that TN induction of *uggt-1* expression is completely abolished *in ire-1*, but remains unaltered in *atf-6* and *pek-1*. These experiments demonstrate a role for *ire-1* in transcriptional regulation of *uggt-1* under TN stress.

### CeUGGT-1 is expressed in the nervous system

**Figure 8:**
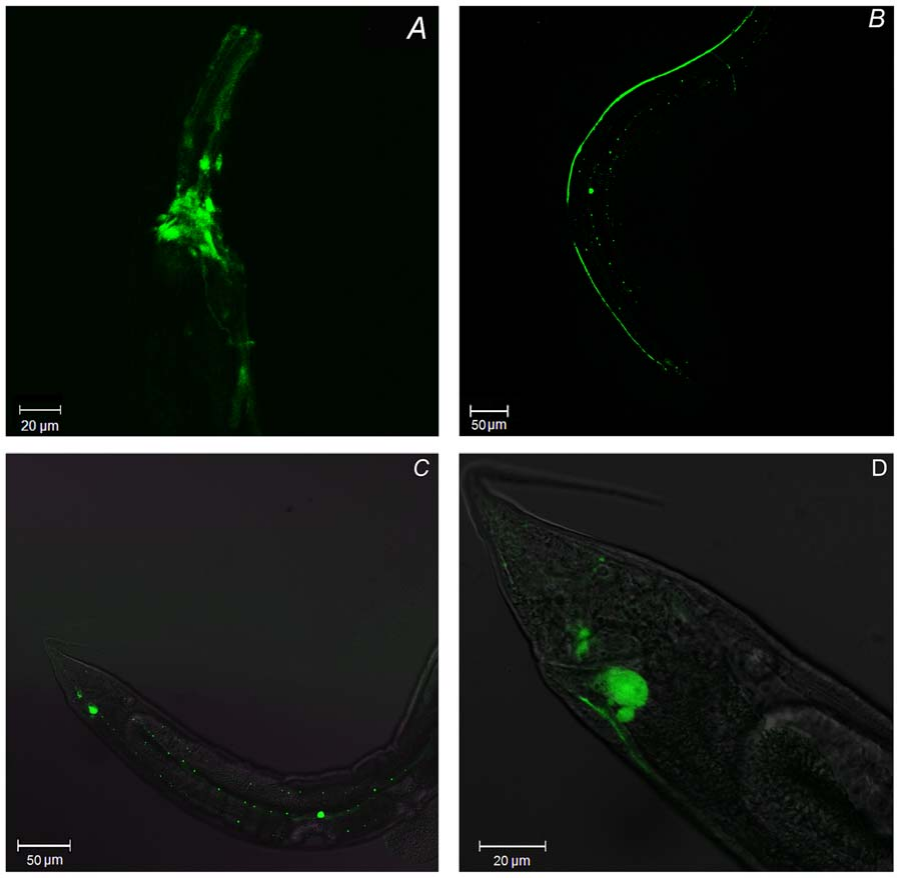
UGGT-1 is expressed in cells of the nervous system. N2 transgenic worms expressing GFP under the control of *uggt-1* promoter were placed in agarose pads and visualized by fluorescence confocal microscopy. A) Amphid neurons of the head and nerve ring, B and C) Neurons in the dorsal and ventral nerve cord and neurons along the body, D) phasmid neurons located at the lateral side of the tail.

To analyze the CeUGGT-1 and CeUGGT-2 body expression pattern we used a reporter gene in which the *uggt-1* and *uggt-2* promoters were fused to GFP coding sequence. We detected GFP expression in larvae and adults transgenic worms containing the *uggt-1::gfp* construct in cells of the nervous system, amphid neurons of the head and the nerve ring (Figure 8A); neurons in the dorsal and ventral nerve cords and neurons along the body (Figure 8B,C). The phasmid neurons located at the lateral side of the tail (Figure 8D). The fact that no signals were found in worms carrying the *uggt-2::gfp* fusion might reflect the fact that the reporter construct, which lacks the introns and 3′untranslated regions, could be missing sequences critical for expression or, alternatively, to the extremely low level of expression of this gene (at most 3% of *uggt-1*). We also analyzed the body pattern expression of these reporters under ER stress (5 µg/ml TN for 6 h) and were unable either to observe an increase in the level of GFP expression in the nervous system or to detect GFP expression in other tissues. Similar results were obtained previously for the body expression pattern of GFP in worms containing an *uggt-1::gfp* construct. Moreover, it was also not possible to observe an increase in GFP expression neither in transgenic worms containing *uggt-1::gfp* nor in *cnx::gfp* constucts treated with TN under the same conditions. [22]. This fact represents an apparent contradiction but differences in GFP expression upon TN treatment may result from a higher sensitivity/accessibility of certain organs/cell types to the drug and also the relative sizes of the organs, whereas real time PCR is an extremely sensitive analytical method that shows the increase in *uggt-1* transcription level in worms as a whole. We may conclude then that the *uggt-1* is mainly expressed in the nervous system but we can not discard the possibility that this gene might be expressed at lower levels in other tissues that are not able to be detected using this reporter. It has been previously mentioned for the *uggt-2::gfp* fusion, that the reporter construct lacks the introns and 3′untranslated regions and could be missing sequences critical for optimal expression.

### CeUGGT-1 depletion causes a reduced lifespan and that of CeUGGT-1 and CeUGGT-2 a delay in development

**Figure 9:**
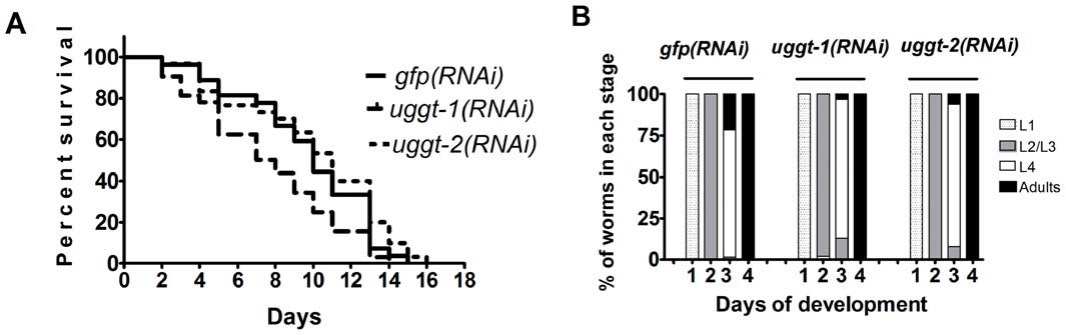
CeUGGT-1 depletion causes a reduced lifespan and that of CeUGGT-1 and CeUGGT-2 a delayed development. F2 *uggt-1(RNAi*) *uggt-2(RNAi)* and control *gfp (RNAi)* worms were seeded on RNAi plates and monitored for survival at 24 h intervals for the next 16 days (A). The survival analysis was performed using the Kaplan-Meier method and the survival curves were compared using the logarithmic rank test. Development to progressive larval stages (B). Gravid hermaphrodites were transferred to RNAi plates for 6 h to lay eggs and the developmental stages of the worms were analyzed for 5 days at intervals of 24 h and the percentages of L1, L2/L3, L4 and adults were determined. These studies were performed for three independent cohorts (n>60) and the results are representative of triplicate experiments (A) and the mean of the three experiments (B).

To investigate phenotypes linked to *uggt-1 and uggt-2* loss-of-function, we analyzed the impact of CeUGGT-1 and CeUGGT-2 depletion on the lifespan and the appearance of morphological and developmental defects. Since we have determined that CeUGGT-1 is mainly expressed in the nervous system, we performed this functional studies in the *C. elegans lin-15b;eri-1* strain (KP3948) which has an enhanced response to double-stranded RNA including the nervous system [23]. F2 *uggt-1(RNAi*) and *uggt-2(RNAi)* worms were analyzed in these experiments since no noticeable changes were detected in the P0 and F1 progeny. Synchronized F2 *uggt-1(RNAi)* and *uggt-2(RNAi)* L1 worms were seeded on RNAi plates and monitored for survival at 24 h intervals for the next 16 days. *gfp(RNAi)* worms were used as controls. The *uggt-1(RNAi)* individuals but not the *uggt-2(RNAi)* ones exhibited a reduced lifespan compared to control worms (median survivals of 7.5 and 10.0 days for the *uggt-1(RNAi)* and either *uggt-2(RNAi)* or controls, respectively (Figure 9A). The logarithmic rank test analysis showed that the *uggt-1(RNAi)* survival curve was different from the *gfp(RNAi)* with a P value <0.05 and no effect on the lifespan was observed in the *uggt-2(RNAi)* individuals (Figure 9A). The development to progressive larval stages in the *uggt-1(RNAi)* and *uggt-2(RNAi)* animals was slightly retarded with respect to controls worms (Figure 9B). The number of *uggt-1(RNAi) and uggt-2(RNAi)* worms that matured to adults was compared to the number of control *gfp(RNAi)* adults worms by day 3 using one-way ANOVA test and Bonferroni's Multiple Comparison Test worms (P value <0.01). This analysis showed that there were significant differences in the number of *uggt-1(RNAi) and uggt-2(RNAi)* adults worms with respect to the control *gfp(RNAi).*

### *uggt-2* is an essential gene and heterozygous *uggt-2 (ok2510)/+* worms show a delay in development

**Figure 10:**
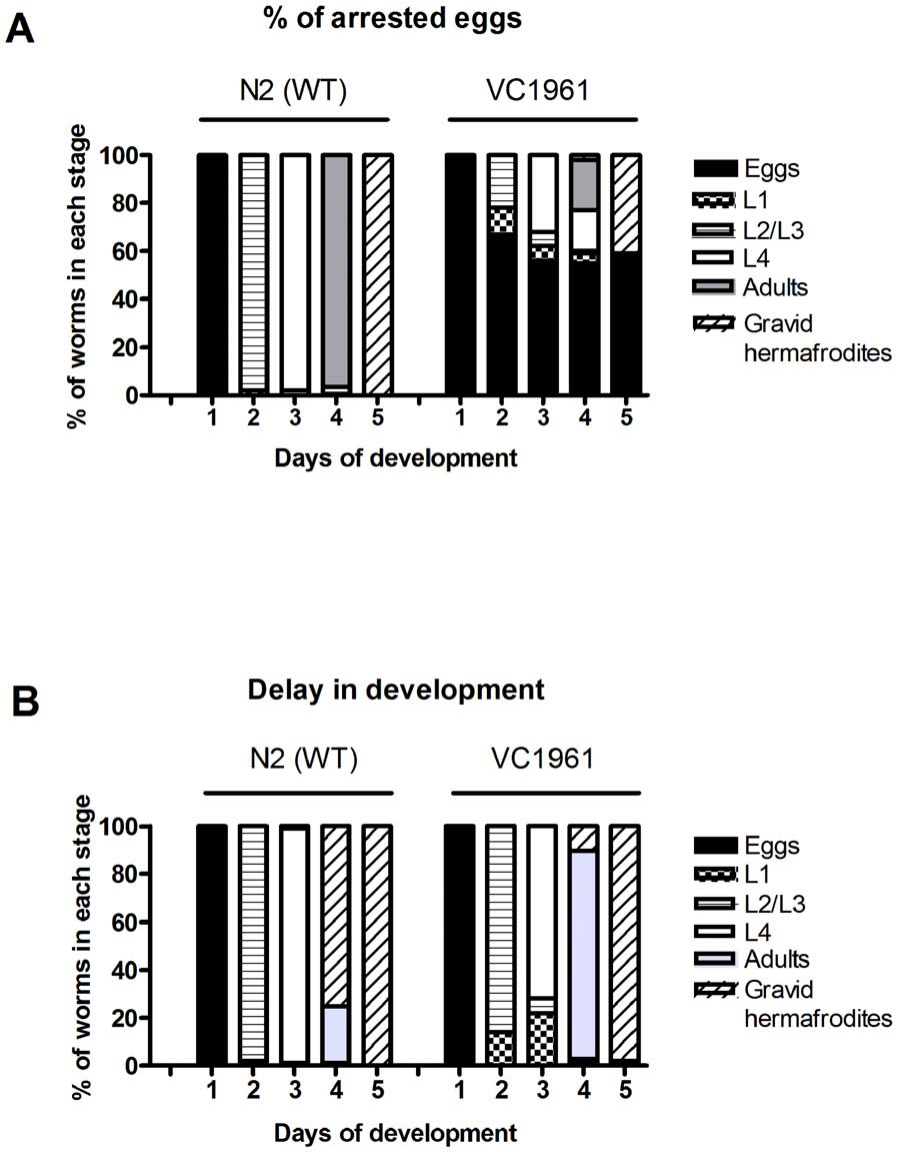
*uggt-2* is an essential gene. Heterozygous *uggt-2 (ok2510*)/+ (VC1961) and N2 wild type gravid hermaphrodites were allowed to lay eggs for 6 h and then removed from plates. Developmental stages were analyzed for 5 d at intervals of 24 h and the percentages of arrested eggs, of L1, L2/L3, L4, adults and gravid hermaphrodites were determined A) Developmental stages were analyzed as in (A) and the percentages of L1, L2/L3, L4, adults and gravid hermaphrodites were determined from hatched eggs. Studies were performed for three independent cohorts (n>200) and the values shown are the mean of the three experiments.

To check previously presented results we cloned individual animals heterozygous for the *ok2510* allele, which carries a 783 bp deletion spanning coding and non coding regions in *uggt-2* gene, from the genetically heterogeneous population. We out-crossed VC1961 heterozygous *uggt-2(ok2510)* worms chromosome balanced with *hT2 (I; III)*, a GFP-marked translocation 4 times to wild-type (N2) nematodes to obtain a clean line VC1961 and N2 wild type gravid hermaphrodites were allowed to lay eggs for 6 h and then removed from plates. Developmental stages were analyzed for 5 d at intervals of 24 h, the number of arrested eggs, and the percentages of eggs, L1, L2/L3, L4, adults and gravid hermaphrodites were determined. The results are shown in Figure 10.

We found that more than 50% of the eggs laid by VC1961 strain were arrested and did not develop to progressive larval stages. Animals that matured to aldulthood were all GFP positive. Since *hT2[qIs48]* is homozygous lethal, all animals expressing GFP are heterozygous for *uggt-2 (ok 2510)*, thus confirming that *uggt-2* is an essential gene as it had been previously proposed (www.wormbase.org).

We also analyzed hatched eggs that matured to progressive larval stages and found that *uggt-2 (ok2510)/+* worms development was slightly retarded with respect to that of wild type worms. We analyzed these results with a 2 tails T-test for one sample and found significant differences between the number of wild type and VC1961 worms that matured to adults by day four (p value 0.018). These results demonstrate that fully expressed CeUGGT-1 activity in this homozygous *uggt-2 (ok2510)/uggt-2 (ok2510)* partial deletion mutant, is not able to overcome the loss of function of CeUGGT-2. On the other hand, the level of expression of *uggt-2* found in this *uggt-2* (ok2510)/+ worms causes a developmental delay similar to that found in the KP3948 *uggt-2 (RNAi)*.

Due to the significant similarity between *uggt-1* and *uggt-2* sequences the possibility that the gene silencing for one of these genes affects the expression of the other may not be eliminated. However, results shown above (Figure 9 panel B) and Figure 10 which show similar effects in development in *uggt-2(RNAi)* and in the heterozygous *uggt-2 (ok2510)/+*, and the fact that no significant decrease in UGGT-1 activity was observed in *uggt-2(RNAi)* worms reinforce the idea that the observed biological effects which are associated to the loss of function of CeUGGT-1 or CeUGGT-2 are not strongly influenced by the silencing of the other gene.

### Both CeUGGT-1 and CeUGGT-2 play a protecting role under ER stress conditions

**Figure 11:**
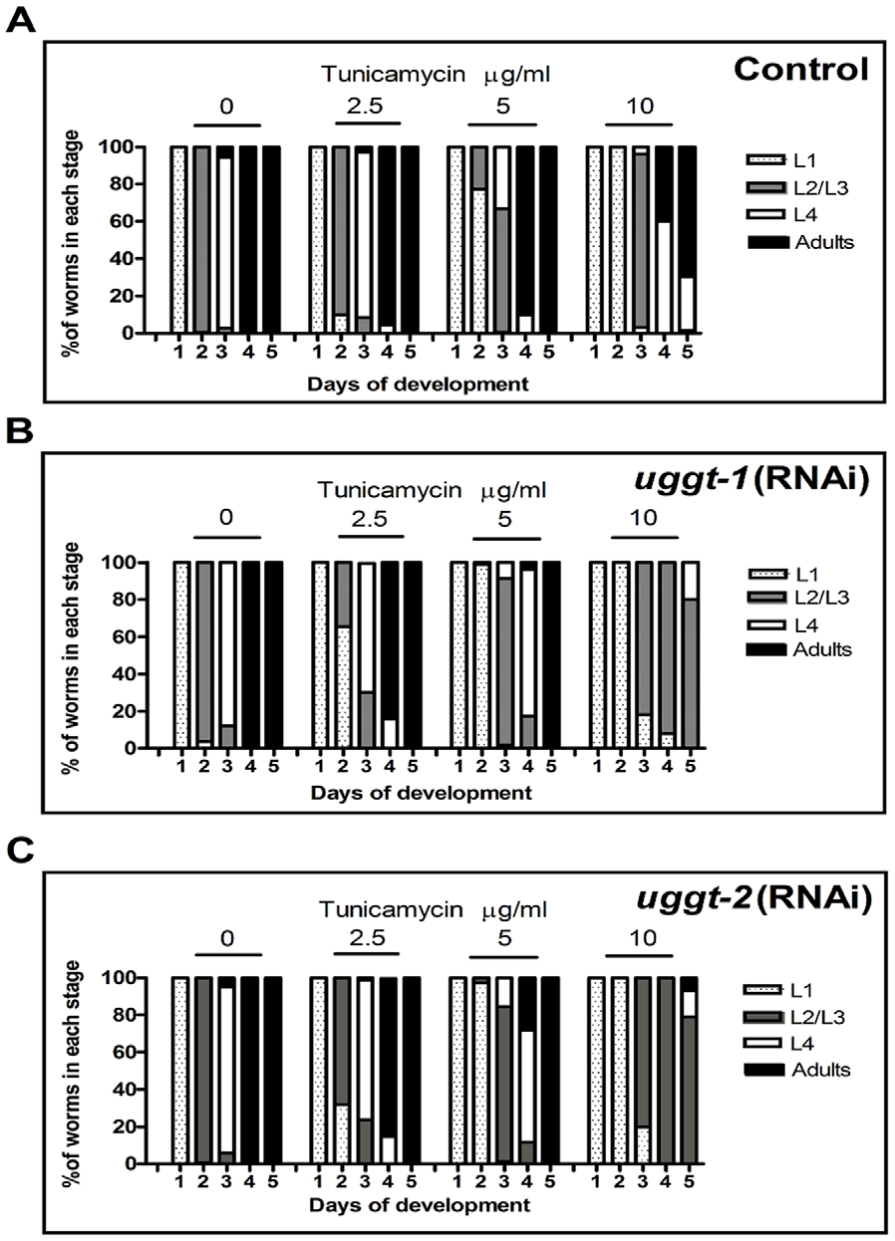
TN sensitivity assay. F2 Control *gfp(RNAi)* (A), *uggt-1(RNAi)* (B) and *uggt-2(RNAi)* (C) worms were tested under several TN concentrations (0, 2.5, 5 or 10 µg/ml). Gravid hermaphrodites were allowed to lay eggs for 6 h and the developmental stages were analyzed for 5 days at intervals of 24 h and the percentages of L1, L2/L3, L4 and adults were determined. Studies were performed for three independent cohorts (n>100) and the values shown are the mean of the three experiments.

We have found only subtle phenotypes in *uggt-1(RNAi)* animals, a reduced life span and a delay in the development through the life cycle. Since UGGT activity facilitates interaction of glycoprotein folding intermediates with the lectin chaperones CNX and CRT we hypothesized that the relevance of CeUGGT-1 expression might become more evident when worms encountered stress conditions. TN is an ER stressor, as it inhibits the glycosylation process thereby causing the accumulation of unfolded proteins in the ER. We have shown above that the level of *uggt-1* mRNA transcription increases when worms are treated with 5 µg/ml TN for 6 h at 20°C (Figure 6). We tested the susceptibility of F2 *uggt-1(RNAi), uggt-2(RNAi)* and *gfp(RNAi)* worms to several TN concentrations (0, 2.5, 5 or 10 µg/ml). Gravid hermaphrodites were allowed to lay eggs for 6 h and the developmental stages (percentages of L1, L2/L3, L4 and adults) were analyzed for 5 days at intervals of 24 h (Figure 11). It was previously reported that the growth rate of wild-type (N2) worms was not affected until a 5 µg/ml TN concentration was reached [17], [24], but we found a slight delay in development when using KP3948 strain worms even at 2.5 µg/ml (Figure 11) and the delay increased with TN concentrations. For instance, at 5 µg/ml by day 4, 90% of *gfp(RNAi)* worms reached adulthood while 96% of the *uggt-1 (RNAi)* and 82% *uggt-2 (RNAi)* worms remained at L2/L3 or L4 stages. This data were analyzed using one way ANOVA and Bonferroni posttest analysis and showed significant differences between the number of *uggt-1(RNAi)* and *uggt-2(RNAi)* worms that reached adulthood and *gfp(RNAi) control worms (P value <0.001 and 0.01 respectively).* The effect of TN became more evident at 10 µg/ml concentration, as at this concentration, 80% of *uggt-1(RNAi)* and *uggt-2(RNAi)* worms were arrested at L2/L3 stage and those that matured to L4 stage became very sluggish and sick. None of the *uggt-1(RNAi)* worms and only 6% of *uggt-2(RNAi)* reached adulthood while 70% of the *gfp(RNAi) did so.* These data were also analyzed and showed significant differences between both treated groups and the control *gfp(RNAi)* (P value <0.001 Bonferroni posttest analysis). We also found important differences in the survival of the interfered worms as 48% of the *gfp(RNAi)* remained alive but only 6% *uggt-1(RNAi)* and 8% of *uggt-2(RNAi)* did so by day 5. The statistical survival analysis was performed using the Kaplan-Meier method and the survival curves were compared using the logarithmic rank test. The survival curves of both treated worms differed from the control with a P value *<*0.002. This results indicate that both CeUGGT-1 and CeUGGT-2 protect the worms against the disruption of ER homeostasis and the accumulation of misfolded proteins and since the absence of this protein arrest at the L2/L3 stage, it may be suggested that both CeUGGT-1 and CeUGGT-2 may play a role in dealing with the endogenous ER stress that worms experience during development.

### CeUGGT-2 is relevant for alleviating stress in the absence of *ire-1* UPR signaling pathway

There is a constant low-level stress in the ER that requires a basal UPR response during cell growth, differentiation or physiological responses. Genetic interactions suggested that *ire-1*, *pek-1* and *atf-6* regulate the worm UPR and are required for growth and survival. Defects in ire-1/xbp-1 signaling in the presence of a mutation in either *pek-1* or *atf-6* caused L2 larval arrest, implying that UPR signaling may be particularly important at this stage of development [17], [18]. We decided to investigate if the activity of CeUGGT-1 and CeUGGT-2 was particularly relevant for development in worms with blocked response to UPR. We performed RNAi experiments in worms with inactivated UPR transducers (IRE-1, ATF-6 and PEK-1) in the absence or presence of very low TN concentrations to mimic conditions of low ER stress found by worms under conditions of normal growth.

**Figure 12:**
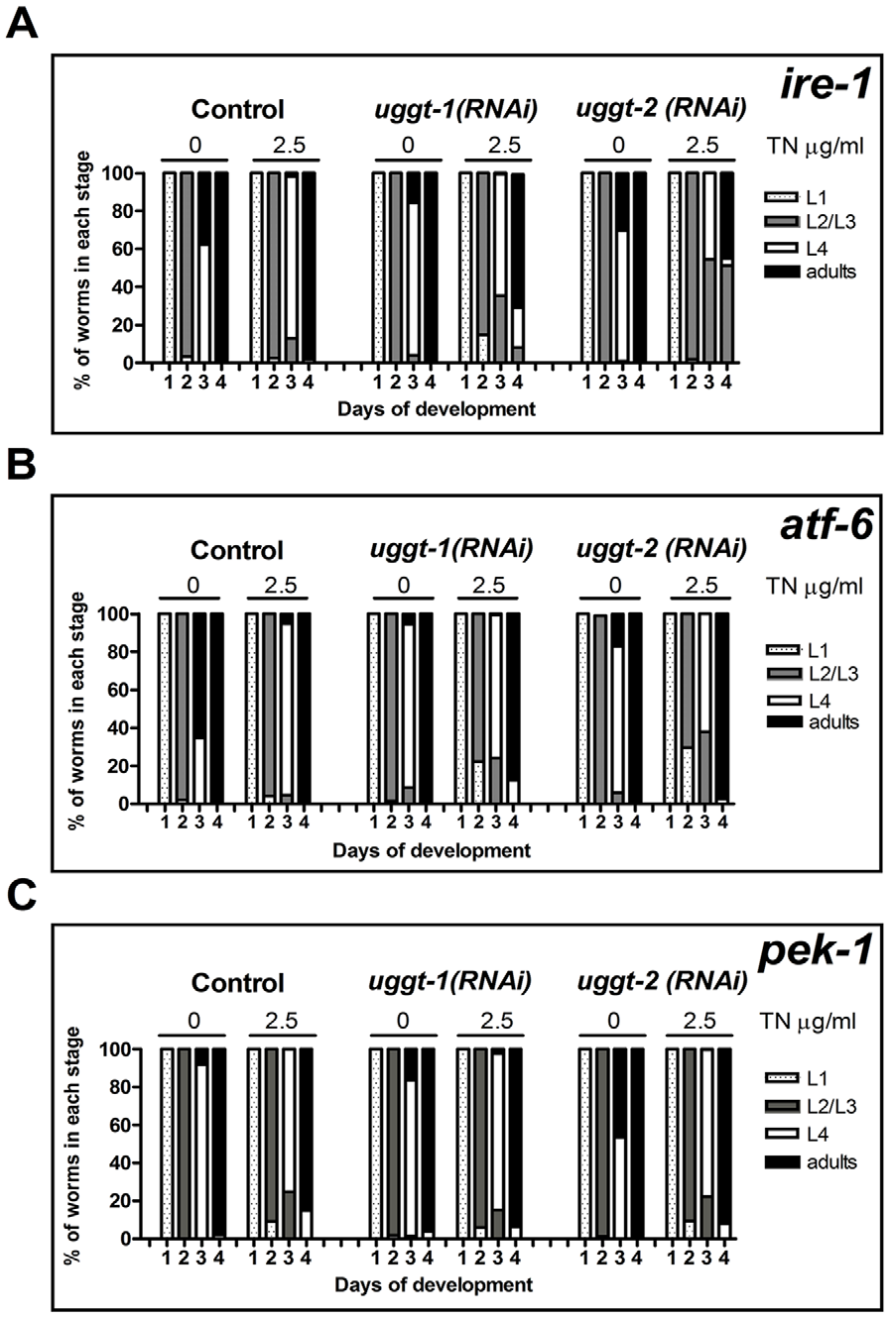
CeUGGT-2 plays a role in alleviating endogenous ER stress during development. F2 Control *gfp(RNAi), uggt-1(RNAi)* and *uggt-2(RNAi)* worms of *ire-1* (A), *atf-6* (B) and *pek-1* (C) strains were tested under 0 and 2.5 µg/ml TN concentrations. Gravid hermaphrodites were allowed to lay eggs for 6 h and the developmental stages were analyzed for 5 days at intervals of 24 h. The percentages of L1, L2/L3, L4 and adults were determined. Studies were performed for three independent cohorts (n>100) and the values are the mean of the three experiments.

*ire-1, uggt-1(RNAi)* worms showed a developmental delay similar to that shown above for KP3948 strain animals. Interestingly *ire-1 uggt-2(RNAi)* worms became sluggish and sick even at 2.5 µg/ml and more than 45% of them were dead by day 4 while 86% of *gfp(RNAi)* remained alive. The survival curves were analyzed as mentioned above and we found significant differences between *uggt-2(RNAi) and the control (P value <0.0001).* More than 50% of *uggt-2(RNAi)* worms that remained alive were arrested at the L2/L3 stage by day 4. These results were analyzed using one way ANOVA test and multiple comparison Bonferroni posttest analysis and differences in development between control *ire-1 gfp(RNAi)* and *ire-1 uggt-2(RNAi)* worms were found to be statistically significant (P value <0.01). It should be noted that a similar developmental difference occurring at a TN concentration of 2.5 µg/ml, was not found for worms with the expression of *uggt-2* interfered but not displaying the *ire-1* mutation or for animals displaying it but with the expression of *uggt-1* gene interfered (Figure 12). It may be suggested, therefore, that CeUGGT-2 is particularly relevant in alleviating endogenous ER stress during development, even though the CeUGGT-1 is fully active. On the other hand, no such differences were found when *atf-6* or *pek-1* worms were studied.

## Discussion

The currently accepted model for the glycoprotein folding quality control mechanism is essentially composed by four proteins, the folding sensor (UGGT), the glycan modifying GII and the monoglucosylated glycan binding lectins CNX and CRT. An additional protein involved is ERp57 a protein oxidoreductase loosely attached to the lectins that catalyzes the correct disulfide bonding in glycoprotein [3]. The genome of *C. elegans* codes for proteins homologous to all participants in the quality control of glycoprotein folding, although not in all cases their role in that mechanism has been confirmed. What we know about the proteins involved in this mechanism is mostly related to CNX and CRT. Each of the worm lectins is encoded by a single gene (*cnx-1 and crt-1*), which are 58% and 38% identical and 71% and 56% similar to their human counterparts at the amino acid level, respectively [24], [25], [26]. CNX knock-out mice survived up to 4 weeks after birth and showed very obvious motor disorders revealing that CNX might be important for neuronal function [27]. CRT knock-out mice showed embryonic lethality along with heart development defects, indicating that CRT is essential for embryogenesis in mammals [28]. In marked contrast, *C. elegans cnx-1 and crt-1* null mutants are viable and showed only mild phenotypic defects: the *cnx-1* null mutant displayed temperature-sensitive developmental defect and retarded growth under stress and the *crt-1* null mutant had a temperature-dependent reproduction defect [25], [26]. A *cnx-1 crt-1* null double mutant was viable and exhibited an additive effect indicating that functions of CNX and CRT are not redundant but only partially overlapping in the worm [26]. The *C. elegans* genome has three conserved PDI encoding genes, namely *pdi-1*, *pdi-2* and *pdi-3*. Recombinant expression demonstrates that all three PDIs are active disulfide isomerases but it has not been clearly determined which of them is the functional homologue of ERp 57 that associates with CNX/CRT. Studies on ERp57-deficient mice also indicate that the protein is critical during embryonic development [29]. Although in the *C. elegans* genome there are ORFs encoding proteins that share a high degree of similarity with both GII subunits of other organisms, neither the ORF that codes for GIIα nor that for GIIβ have been confirmed to be the codes for the proteins involved in the initial stages of glycan processing.

The last component of this mechanism, the UGGT, has not been studied to date in *C. elegans.* In this report we show that *C. elegans* displays indeed an UGGT activity. As we have mentioned above, there are genes coding for UGGT-like proteins only in Eutelostomi and in some members of the genus *Caenorhabditis* belonging to the phylum Nematoda that have arisen from two independent gene duplications. In this report we show that ORF F48E3.3 encodes an active UGGT. The protein appeared to be upregulated under ER stress, similarly to what has been found for the human and *S. pombe* UGGTs. CeUGGT-1 is expressed in the nervous system and its expression pattern partially overlapped with that found for CNX and CRT. The former lectin is in addition expressed in the H-shaped excretory cells and in the spermatheca [26] and CRT is broadly expressed in many other tissues as intestine, uterus, pharynx and body wall muscle [30]. The fact that CeUGGT-1 is expressed in cells of the nervous system may reflect the requirement of neuron cells to deal with the dramatic increase in membrane glycoprotein synthesis both during differentiation and growth. It has been previously reported that in mammals neurons have to handle an endogenous UPR during development [31], [32]. We have found that CeUGGT-1 is expressed during the entire life cycle with a maximum expression at larval stages and that depletion of CeUGGT-1 causes both a reduction in lifespan and a delay in maturing to progressive larval stages during development. These findings are consistent with the idea that the expression of proteins that are induced when the ER homeostasis is disrupted by extrinsic factors are indeed required for normal development in *C. elegans* even in the absence of stress [33]. Moreover, our results are supported by the fact that when *uggt-1(RNAi)* treated worms were subjected to ER stress produced by a TN treatment that induced hypoglycosylation and accumulation of misfolded proteins, they arrested development at the L2/L3 stage by day 5, whereas under the same conditions mock treated worms overcame the stress and matured to adulthood.

We also undertook the study of the biological role of CeUGGT-2, the other UGGT-like protein encoded in *C. elegans* genome. We first checked if it also displayed UGGT activity using the same expression system that we had used for UGGT-1 (a *uggt-2* codon-usage optimized version in a *S. pombe* mutant lacking the activity, see Materials and Methods), but, although both *uggt-1* and *uggt-2* were similarly transcribed and that both optimized genes had a similar frequency of optimal codons (86–87%), we were unable to detect an enzymatic activity in yeast microsomes that specifically glucosylated denatured glycoprotein acceptors or that *in vivo* created monoglucosylated folding intermediates and misfolded glycoproteins. We also measured UGGT activity in microsomes of *uggt-2(RNAi)* worms and found activity levels similar to those of mock treated animals, whereas microsomes of *uggt-1(RNAi)* worms revealed a significant reduction (almost 60%) in UGGT activity (Figure 4). Although this result may reflect the low transcription level of *uggt-2*, it also supports the idea that CeUGGT-2 lacks UGGT activity. However, as we have been unable to demonstrate the presence of CeUGGT-2 in *S. pombe* cells, the enzymatic activity of this protein is still an open question. On the other hand, the fact that UGGT-1 is not able to replace the loss of function of UGGT-2 in a homozygous *uggt-2/uggt-2* partial deletion mutant, reinforce the idea that these proteins display different activities. Moreover, we confirmed that *uggt-2* is an essential gene thus confirming that although *uggt-2* is transcribed at a very low level it is not actually a pseudogene.

As in other vertebrates, there are two UGGT homologues expressed in human cells, HUGT-1 and HUGT-2, that share a 55% identity between them (83% and 49% in the C- and the N-terminal domains, respectively). Whereas HUGT-1 and HUGT-2 colocalized to the ER, the former but not the latter displayed UGGT activity and was upregulated under ER stress conditions [8]. A chimera containing HUGT-1 N-terminal and HUGT-2 C-terminal domains was enzymatically active but the role of HUGT-2 is still unknown [34]. Our findings concerning the glucosyltransferase activity of both *C. elegans* UGGT homologues are similar, therefore, to those found for the human proteins HUGT-1 and HUGT-2 [8]. CeUGGT-1 shares a higher degree of similarity with UGGTs from organisms encoded by single genes (35–42%) than CeUGGT-2 (32–35%) according to the Clustal W sequence analysis program (Figure S3). On the other hand CeUGGT-1 and CeUGGT-2 share the same degree of similarity to HUGT-1 and HUGT-2 although the parameter is higher for CeUGGT1 (41–42%) than CeUGGT-2 (34–36%). It seems that CeUGGT-1 and CeUGGT-2 arose from a gene duplication event independent from that occurred in Eutelostomi and while CeUGGT-1 plays a role as a sensor of glycoprotein conformations CeUGGT-2 has diverged to gain another biological function.

The different phenotypes associated to the loss of function of CeUGGT-1 and CeUGGT-2 reinforce the idea that they play different biological roles. While the lifespan analysis of *uggt-1(RNAi)* worms showed a significant difference with that of mock treated control animals, that of *uggt-2(RNAi)* worms did not. Furthermore, the delay in reaching progressive larval stages was less significant for *uggt-2(RNAi)* than for *uggt-1(RNAi)* worms and *uggt-2* expression was not upregulated under ER stress conditions. We found that CeUGGT-2 was also needed for development since *uggt-2(RNAi)* worms, the same as *uggt-1(RNAi)* ones, arrested development at the L2/L3 stage when subjected to ER stress. However, when we depleted CeUGGT-1 and CeUGGT-2 in *C. elegans ire-1* mutant strain with one UPR signaling pathway a blocked and treated them with a very low TN concentration, we found a different outcome. Interestingly, *ire-1 uggt-2(RNAi)* worms became sluggish and sick; half of them were dead by day 4 and 50% of those that survived stopped development at the L2/L3 stage while no such effect was observed in *ire-1 uggt-1(RNAi)* or *ire-1 gfp(RNAi)* worms confirming that both UGGT homologues play different roles. The differential function of CeUGGT-2 became, therefore, more evident in cells unable to cope with the altered ER homeostasis. Since *ire-1* worms are unable to transduce signals that would produce the synthesis of proteins engaged in alleviating the ER stress, homeostasis cannot be restored and lack of CeUGGT-2 arrested worms in early development or caused general defects that generated death. Under the same conditions lack of CeUGGT-1 expression produced the same effect occurring in worms with the three active ER stress transducers, thus showing that CeUGGT-1 and CeUGGT-2 play different roles in the cell. It may be speculated that CeUGGT-2 is involved in alleviating the broken ER homeostasis that metazoans cells must overcome during development through another pathway. Although the lack of functional replacement mentioned above may reflect the possible expression of both proteins in different tissues, the fact that *cnx/crt* but not *uggt-2* null mutants are viable indicates that the biological role of CeUGGT-2 is not dependent on the chaperone-lectin as canonical UGGTs are.

On the other hand several of the different results found for CeUGGT-1 and CeUGGT-2 may be explained by a possible protein expression in different tissues. The most important evidence that both proteins display different functions is that *uggt-2* proved to be an essential gene, whereas worms showing less than 42% of the activity present in wild type worms only show mild phenotypes. *C. elegans* provides an excellent genetic model for the study of the biological function of the UGGT homologue lacking a canonical glucosyltransferase activity (CeUGGT-2) and also is an appropriate system for studying the role of both proteins in diseases related to protein folding.

## Supporting Information

Figure S1 **pH and cation dependence of UGGT activity in** ***C. elegans.*** N2 wild type worm microsomal proteins were incubated in a mixture that contained A) 10 mM CaCI_2_, 0.6% Triton X-100, 5 mM NMDNJ and 3 µCi UDP-[^14^C]Glc, at 20°C for 30 min with 20 mM concentration of the indicated buffers -•-•- imidazole HCl; -○-○- Tris HCl. B) 20 mM Tris-HCl pH 7.5, 0.6% Triton X-100, 5 mM NMDNJ and 3 µCi UDP-[^14^C]Glc, at 20°C for 30 min and -•-•-CaCI_2_, -○-○- MgCl_2_ and -▾-▾ MnCl2 in the indicated concentrations. Reactions were stopped with 1 ml of 10% of trichloroacetic acid. After centrifugation, the pellets were twice washed with 1 ml of 10% trichloroacetic acid and counted. The values shown are the mean of two independent experiments. Error bars represent standard deviations.
(TIF)

Figure S2 ***uggt-1*** **but not** ***uggt-2*** **is upregulated under stress conditions in L2/L3 stage.** Total RNA from untreated and 5 µg/ml TN-treated L2/L3 animals was isolated and the levels of *uggt-1*, *uggt-2* and *hsp4* expression were quantified by real-time PCR using *ama-1* as reference gene. Relative expression level represent RNA expression in TN treated worms/RNA expression in untreated worms. The value obtained for untreated samples was considered as one. The values shown are the mean of three independent experiments. Error bars represent standard deviations * indicates significant differences.
(TIF)

Figure S3 **Sequence alignment of HUGT-1, HUGT2, CeUGGT-1 and UGGT-2 by the Clustal W program.** Consensus symbols used by Clustal W are: (*) means that the residues in that column are identical in all sequences in the alignment, **(:)** means that conserved substitutions have been observed, **(•)** means that semi-conserved substitutions are observed.
(PDF)
