# Supplementary figures and images for: The Two Caenorhabditis elegans UDP-Glucose:Glycoprotein Glucosyltransferase Homologues Have Distinct Biological Functions
Source: PLoS One. 2011 Nov 2;6(11):e27025. doi: 10.1371/journal.pone.0027025 (PMC3206904; doi:10.1371/journal.pone.0027025)

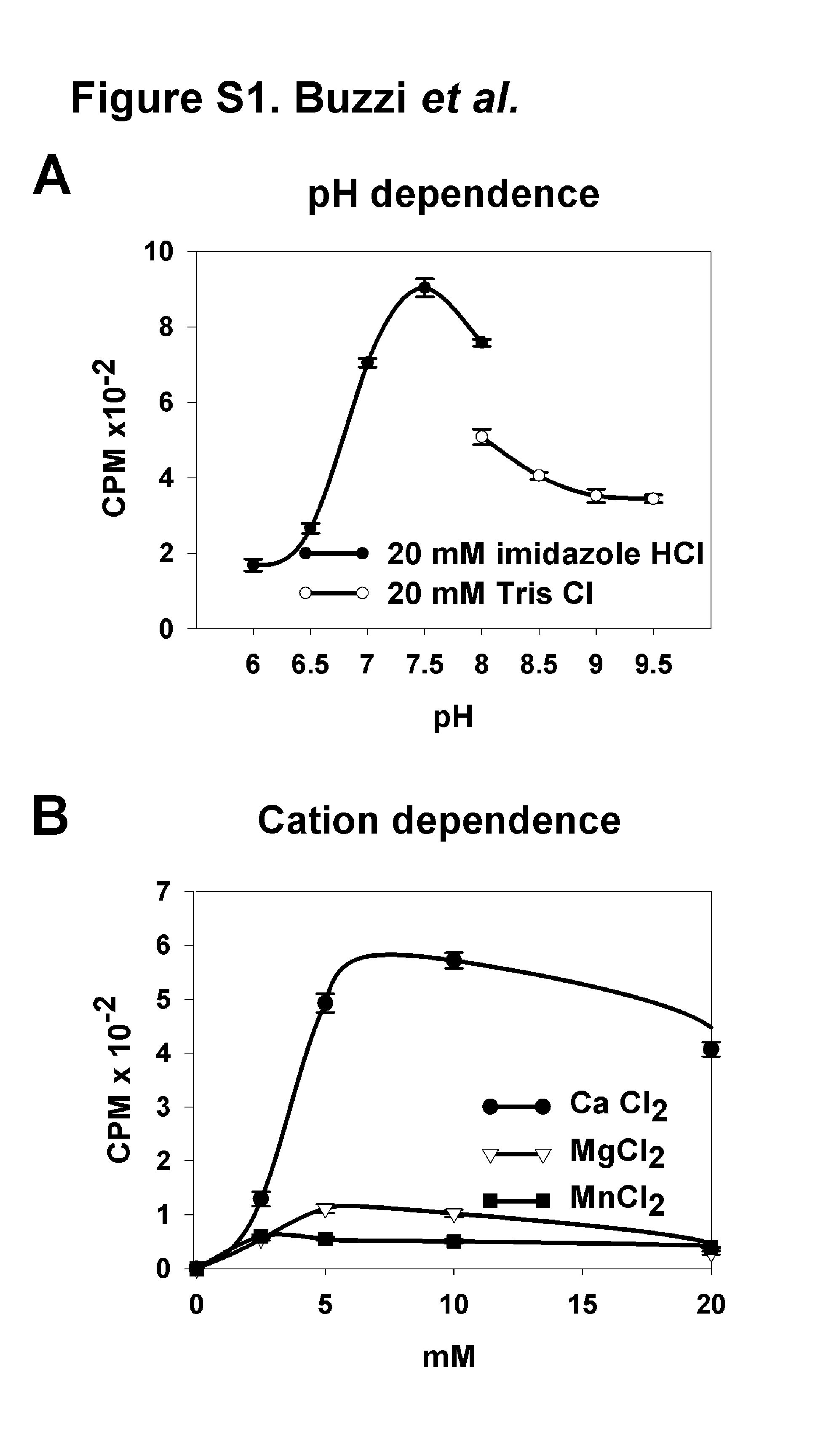

Supplement: Figure S1 — pH and cation dependence of UGGT activity in C. elegans. N2 wild type worm microsomal proteins were incubated in a mixture that contained A) 10 mM CaCI2, 0.6% Triton X-100, 5 mM NMDNJ and 3 µCi UDP-[14C]Glc, at 20°C for 30 min with 20 mM concentration of the indicated buffers -•-•- imidazole HCl; -○-○- Tris HCl. B) 20 mM Tris-HCl pH 7.5, 0.6% Triton X-100, 5 mM NMDNJ and 3 µCi UDP-[14C]Glc, at 20°C for 30 min and -•-•-CaCI2, -○-○- MgCl2 and -▾-▾ MnCl2 in the indicated concentrations. Reactions were stopped with 1 ml of 10% of trichloroacetic acid. After centrifugation, the pellets were twice washed with 1 ml of 10% trichloroacetic acid and counted. The values shown are the mean of two independent experiments. Error bars represent standard deviations. (TIF) [file pone.0027025.s001.tif]

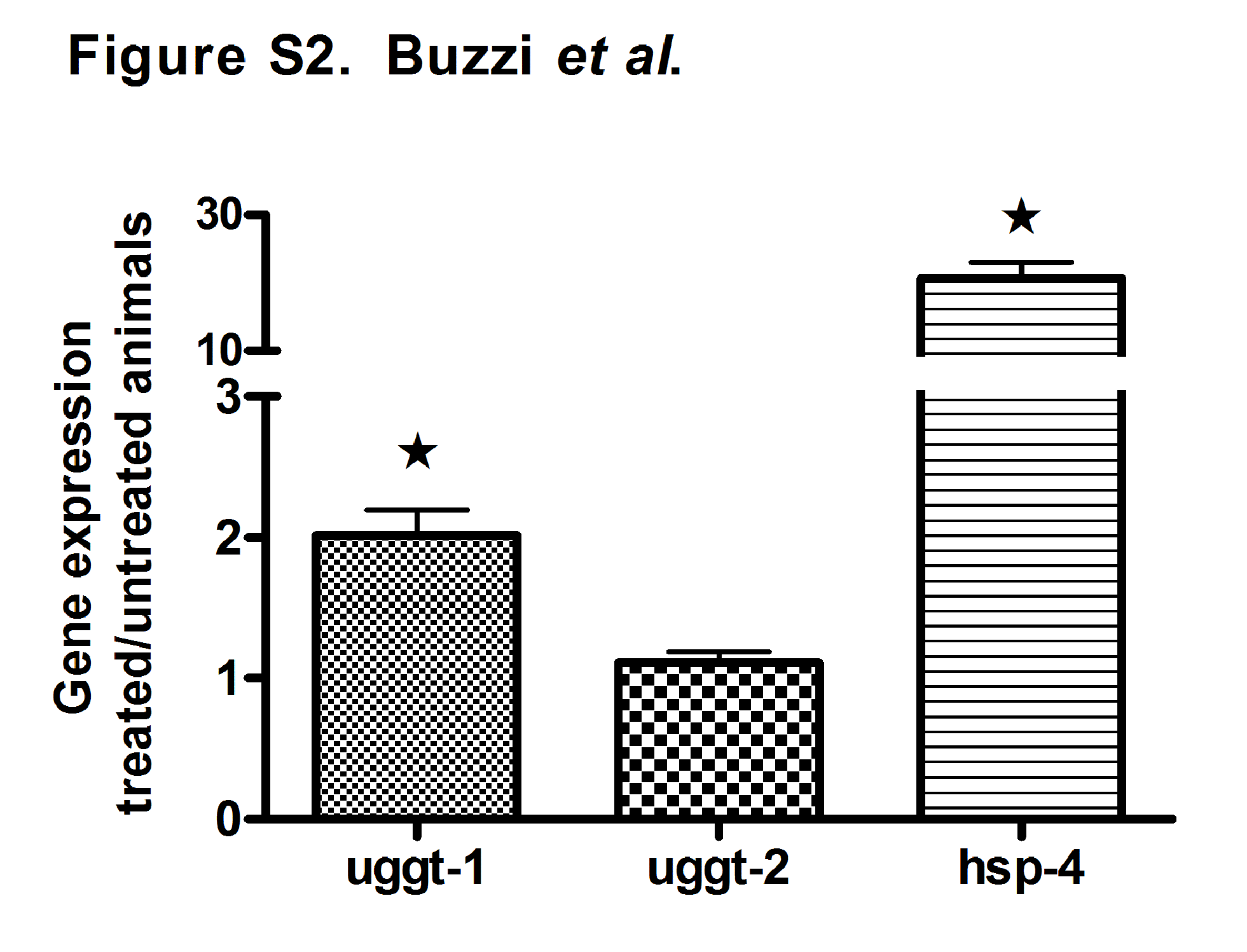

Supplement: Figure S2 — uggt-1 but not uggt-2 is upregulated under stress conditions in L2/L3 stage. Total RNA from untreated and 5 µg/ml TN-treated L2/L3 animals was isolated and the levels of uggt-1, uggt-2 and hsp4 expression were quantified by real-time PCR using ama-1 as reference gene. Relative expression level represent RNA expression in TN treated worms/RNA expression in untreated worms. The value obtained for untreated samples was considered as one. The values shown are the mean of three independent experiments. Error bars represent standard deviations * indicates significant differences. (TIF) [file pone.0027025.s002.tif]
