# Supplementary material for: The Two Caenorhabditis elegans UDP-Glucose:Glycoprotein Glucosyltransferase Homologues Have Distinct Biological Functions
Source: PLoS One. 2011 Nov 2;6(11):e27025. doi: 10.1371/journal.pone.0027025 (PMC3206904; doi:10.1371/journal.pone.0027025)
Supplement: Figure S3 — Sequence alignment of HUGT-1, HUGT2, CeUGGT-1 and UGGT-2 by the Clustal W program. Consensus symbols used by Clustal W are: (*) means that the residues in that column are identical in all sequences in the alignment, (:) means that conserved substitutions have been observed, (•) means that semi-conserved substitutions are observed. (PDF) [file pone.0027025.s003.pdf]

Figure S3 Buzzi et al.

```
                                   10        20        30        40        50
                         ....|....|....|....|....|....|....|....|....|....|
HUGT1                    MGCKGDASGACAAGALPVTGVCYKMGVLVVLTVLWLFSSVKADSKAITTS 50
HUGT2                    ------------MAPAKATNVVRLLLGSTALWLSQLGSGTVAASKSVTAH 38
CeUGGT1                  ---------------MNLTGLLIFFCHIAVLAALEK--------KGVHTS 27
CeUGGT2                  ---------------MRLTHFLLFLANLKFCWNDNS----------INVR 25
Clustal Consensus                          * .   :                     : .

                                   60        70        80        90       100
                         ....|....|....|....|....|....|....|....|....|....|
HUGT1                    LTTKWFSTPLLLEASEFLAEDSQEKFWNFVEASQNIG--SSDHDGTDYSY 98
HUGT2                    LAAKWPETPLLLEASEFMAEESNEKFWQFLETVQELA--IYKQTESDYSY 86
CeUGGT1                  LKANWDSTSLLAEASEFIAEENEKLFVKFIDIVNKDVGTLNWEKLTDEQK 77
CeUGGT2                  LNAKWRSSSILAEISEYIGKENPEAFWNFVNSVNQEIDETSNKNDSIRNK 75
Clustal Consensus        * ::* .:.:* * **::.::. : * :*::  ::       .  :  . 26

                                  110       120       130       140       150
                         ....|....|....|....|....|....|....|....|....|....|
HUGT1                    YHAILEAAFQFLSPLQQNLFKFCLSLRSYSATIQAFQQIAADEPPPEGCN 148
HUGT2                    YNLILKKAGQFLDNLHINLLKFAFSIRAYSPAIQMFQQIAADEPPPDGCN 136
CeUGGT1                  YEYTIKTAGKVLSTSSVDLLKFALALRQYSPRVQSFQQIAVEYG--EKCD 125
CeUGGT2                  YDFGMSVANNILGPFEMKLLRFSLATRSISPRVQAHEQIGMEFKPEKCSF 125
Clustal Consensus        *.  :. * :.*.    .*::*.:: *  *. :* .:**. :    . . 45

                                  160       170       180       190       200
                         ....|....|....|....|....|....|....|....|....|....|
HUGT1                    SFFSVHGKKTCESDTLEALLLTASERPKPLLFKGDHRYPSSNPESPVVIF 198
HUGT2                    AFVVIHKKHTCKINEIKKLLKKAASRTRPYLFKGDHKFPTNKENLPVVIL 186
CeUGGT1                  VFVVVGEQVSCEYTKLEKMIKDAKTN--SQVLESDHIFG-EKDLKQAAIL 172
CeUGGT2                  SFFVYGGQAGCQLSELN---LNNYDDNNLKIFSFDHIYPVQSTAEKTILI 172
Clustal Consensus         *.    :  *:   ::             ::. ** :  ..    . ::

                                  210       220       230       240       250
                         ....|....|....|....|....|....|....|....|....|....|
HUGT1                    YSEIGSEEFSNFHRQLISKSNAGKINYVFRHYIFNPRKEPVYLSGYGVEL 248
HUGT2                    YAEMGTRTFSAFHKVLSEKAQNEEILYVLRHYIQKPSSRKMYLSGYGVEL 236
CeUGGT1                  YGELGTTSFAKAWEKLS---KLQKTKLIFRHFSKKTDSHPVSLSGYGVEL 219
CeUGGT2                  YGELGTYELKNLLLEAQSLIEKHKNLRFVVRHFSRFSDQKPSLSGYGVEL 222
Clustal Consensus        *.*:*:  :           :  :   .. :.  .  ..   ******** 75

                                  260       270       280       290       300
                         ....|....|....|....|....|....|....|....|....|....|
HUGT1                    AIKSTEYKAKDDTQVKGTEVNTTVIGENDPIDEVQGFLFGKLRDLHPDLE 298
HUGT2                    AIKSTEYKALDDTQVK--TVTNTTVEDETETNEVQGFLFGKLKEIYSDLR 284
CeUGGT1                  AIKNTEYKAVDESSEK------KNVEEDE--ADLFGFNIKLLKELHPDSV 261
CeUGGT2                  ALKNTEYKAVDSSSTN----------KLDEPENLHGLNFKILKNRHLELQ 262
Clustal Consensus        *:*.***** *.:. :          .     :: *: :  *:: : :   96

                                  310       320       330       340       350
                         ....|....|....|....|....|....|....|....|....|....|
HUGT1                    GQLKELRKHLVESTNEMAPLKVWQLQDLSFQTAARILASPVELALVVMKD 348
HUGT2                    DNLTAFQKYLIESNKQMMPLKVWELQDLSFQAASQIMSAPVYDSIKLMKD 334
CeUGGT1                  DAIESFRVNLKESD-ELTPLKRWELQDLSYQAAQKIVNAGPADAIGTLEE 310
CeUGGT2                  NELESLRENLEKQG-EIVPLKQWQLKDLGFKTCQKLKS---LTEIREMEI 308
Clustal Consensus        . :  ::  * :.  :: *** *:*:**.:::. ::        :  ::  120

                                  360       370       380       390       400
                         ....|....|....|....|....|....|....|....|....|....|
HUGT1                    LSQNFPTKARAITKTAVSSELRTEVEENQKYFKGTLGLQPGDSALFING- 397
HUGT2                    ISQNFPIKARSLTRIAVNQHMREEIKENQKDLQVRFKIQPGDARLFING- 383
CeUGGT1                  YSQNFPTHARALAKTSVSDLLRKEVLQNRK-MLEKASIEVGETSLYING- 358
CeUGGT2                  LLQDFPTHARVTSHQNLNETLQRSIQKGQETLEAKG-IESGTNILAINGR 357
Clustal Consensus          *:** :**  ::  :.. :: .: :.:: :     :: *   * ***

                                  410       420       430       440       450
                         ....|....|....|....|....|....|....|....|....|....|
HUGT1                    -LHMDLDTQDIFSLFDVLRNEARVMEGLHR-------LGIEGLSLHNVLK 439
HUGT2                    -LRVDMDVYDAFSILDMLKLEGKMMNGLRN-------LGINGEDMSKFLK 425
CeUGGT1                  -INQDINSLDLFKLADLLKQENKLADGFHS-------MGINREYLSILVG 400
CeUGGT2                  VIAKGDSFVDLFALMEKVEEEKKMVNEFVKGFGNSETEEFERINIPKMLT 407
Clustal Consensus         :  . .  * * : : :. * :: : :           ::   :  .:

                                  460       470       480       490       500
```

```
                         460       470       480       490       500
                  ....|....|....|....|....|....|....|....|....|....|
HUGT1             LN-IQPSE-ADYAVDIRSPAISWVNNLEVDSRYNSWPSSLQELLRPTFPG 487
HUGT2             LN-SHIWE-YTYVLDIRHSSIMWINDLENDDLYITWPTSCQKLLKPVFPG 473
CeUGGT1           MD-TSDDEKTTYAVDHREGYPFFINNLDTDKKYKQWGNSVKLMLQPYYPG 449
CeUGGT2           LVDLSSVKLSEHAFDYSIAEPVYLNDLES---TRSPYKSLMLMLQPFPPG 454
Clustal Consensus :      :   :..*       ::*:*:         .*   :*:*  ** 176

                         510       520       530       540       550
                  ....|....|....|....|....|....|....|....|....|....|
HUGT1             VIRQIRKNLHNMVFIVDPAHETTAELMNTAEMFLSNHIPLRIGFIFVVND 537
HUGT2             SVPSIRRNFHNLVLFIDPAQEYTLDFIKLADVFYSHEVPLRIGFVFILNT 523
CeUGGT1           MIRPIARNLFSLVFVVDPSTSEGRKFLRIGQTFNSHDIAMRIGYIFAVNQ 499
CeUGGT2           QIRPISRNIFNLIMFLDPFDSDDRVFDDVIRNFQTG-IHIRFGFVPILDE 503
Clustal Consensus  :  * :*:..:::.:**  .    :      * :  : :*:*::  ::

                         560       570       580       590       600
                  ....|....|....|....|....|....|....|....|....|....|
HUGT1             SEDVDGMQDAGVAVLRAYNYVAQEVDDYHAFQTLTHIYNKVRTG-EKVKV 586
HUGT2             DDEVDGANDAGVALWRAFNYIAEEFDISEAFISIVHMYQKVKKDQNILTV 573
CeUGGT1           DTKASGETDLGVALLNLFNFVSIDSSNADALKVLNNFLDDYRSK--DPTI 547
CeUGGT2           AKYG---------------------------------------------- 507
Clustal Consensus

                         610       620       630       640       650
                  ....|....|....|....|....|....|....|....|....|....|
HUGT1             EHVVSVLEKKYPYVEVNSILGIDSAYDRNRKEARGYYEQTGVGPLPVVLF 636
HUGT2             DNVKSVLQNTFPHANIWDILGIHSKYDEERKAGASFYKMTGLGPLPQALY 623
CeUGGT1           EDIKEFFEAKFSDASFSDVFGVNSDYDKGRKHGFEFVQKTGLNSAPKVLL 597
CeUGGT2           KSIEEAVDSMIVPPTKKVFWKSKDSLINALKKGSRFVAEAGLTQLPLVLL 557
Clustal Consensus . : . .:          .   ..   .  * .  :   :*:   * .*  208

                         660       670       680       690       700
                  ....|....|....|....|....|....|....|....|....|....|
HUGT1             NGMPFEREQLDPDELETITMHKILETTTFFQRAVYLGELPHDQDVVEYIM 686
HUGT2             NGEPFKHEEMNIKELKMAVLQRMMDASVYLQREVFLGTLNDRTNAIDFLM 673
CeUGGT1           NGFILDDEGVRGDNIEETIMMEVMKISPKIQRAIMEGKLTDRMNVGNWVL 647
CeUGGT2           NGYPLDVTSIER--FESSLTQNIQKQTTRLQLALLHGLIEDSVQIDRWWF 605
Clustal Consensus **  :.   :    ::     .: . :  :*  :  * : .  :   : : 224

                         710       720       730       740       750
                  ....|....|....|....|....|....|....|....|....|....|
HUGT1             NQPNVVPRINSRILTA--ERDYLDLTASNN-FFVDDYARFTILDSQGKTA 733
HUGT2             DRNNVVPRINTLILRT--NQQYLNLISTSVTADVEDFSTFFFLDSQDKSA 721
CeUGGT1           EQKDVMPRINKRILSAPSKKTYVEILGSMDCKSLKDVEN---LSDSDKAG 694
CeUGGT2           EK-----------------KTNPDIIQRLNQRITTAFNNKEFLNISRRSI 638
Clustal Consensus ::                 :   ::                 *. . ::  232

                         760       770       780       790       800
                  ....|....|....|....|....|....|....|....|....|....|
HUGT1             AVANSMNYLTKKGMSSKEIYDDSFIRPVTFWIVGDFDSPSGRQLLYDAIK 783
HUGT2             VIAKNMYYLTQD--------DESIISAVTLWIIADFDKPSGRKLLFNALK 763
CeUGGT1           CLLQTTKYLQKA--------SADSILPVTLWVVADAEAASGRRFIYNSLQ 736
CeUGGT2           QFLKNVHYFSEES------DTVLANADVTTWIIADFENPSNRLFATKAIR 682
Clustal Consensus  . :.  *: :                **  *::.* : .*.* :  .:::

                         810       820       830       840       850
                  ....|....|....|....|....|....|....|....|....|....|
HUGT1             HQKSSNNVRISMINNP-AKEISYENTQISRAIWAALQTQTSNAAKNFITK 832
HUGT2             HMKTSVHSRLGIIYNP-TSKINEENTAISRGILAAFLTQKNMFLRSFLGQ 812
CeUGGT1           ILKNSANSRVGIIFNPESVEKACESNSISSYIRAALDFLPMDQAKRLILK 786
CeUGGT2           SIASQKNNKIALIPNPSSSRSNDNPCSFN------FENVGPNSDMTILSK 726
Clustal Consensus    .. : ::.:* ** : .   :   :.      :          :: :

                         860       870       880       890       900
                  ....|....|....|....|....|....|....|....|....|....|
HUGT1             MAKEGAAEALAAGAD-IAEFSVGGMDFSLFKEVFESSKMDFILSHAVYCR 881
HUGT2             LAKEEIATAIYSGDK-IKTFLIEGMDKNAFEKKYNTVGVNIFRTHQLFCQ 861
CeUGGT1           LSNEEYAADFISGKITFDDLSVGGMDTAKFLADKKKLDCERTRLESQIVK 836
CeUGGT2           IES----------------FCMKNINLSPLDIG----------------- 743
Clustal Consensus : .                : : .::   :                    269
```

```
Clustal Consensus  : .                : : .::   :                     269

                            910       920       930       940       950
                   ....|....|....|....|....|....|....|....|....|....|
HUGT1              DVLKLKKGQRAVISNGRIIGPLEDSELFNQDDFHLLENIILKTSGQKIKS 931
HUGT2              DVLKLRPGEMGIVSNGRFLGPLDED--FYAEDFYLLEKITFSNLGEKIKG 909
CeUGGT1            KVLDISSGGRVVVGNALQVGPLESSEHFEAADFKLLESMLLSRGAEVISS 886
CeUGGT2            ----VNPGETVVVSNGLLIGPLAGRTELLKTDDFNYLDTFWKEKGATKAA 789
Clustal Consensus      :  *   ::.*.  :***     :   *     .   .  .    .

                            960       970       980       990      1000
                   ....|....|....|....|....|....|....|....|....|....|
HUGT1              HIQQLRVEED--VASDLVMKVDALLSAQPKGDPRIEYQFFEDRHSAIKLR 979
HUGT2              IVENMGINAN--NMSDFIMKVDALMSSVPKRASRYDVTFLRENHSVIKTN 957
CeUGGT1            HLKKWEFDVSNGVGSNTVFSIAGHVGKHASSQKRTWVSIQGDEHSVVTLP 936
CeUGGT2            TFFNENTVYD--VTISFYCSIAKKFKEDQQRMDFDEFMESGNGNTIIFPP 837
Clustal Consensus   . :     .     .   .:   .    .           : :: :

                           1010      1020      1030      1040      1050
                   ....|....|....|....|....|....|....|....|....|....|
HUGT1              PKEGE-TYFDVVAVVDPVTREAQRLAPLLLVIAQLINMNLRVFMNCQSKL 1028
HUGT2              PQEND-MFFNVIAIVDPLTREAQKMAQLLVVIGKIINMKIKLFMNCRGRL 1006
CeUGGT1            ADEMDRPAVDVLAVVDPLTMEAQKLGSILHLIKKVTNCEIKIVMNPKDKH 986
CeUGGT2            IDSTN-STITVTWIANPVSREAQQIISVVKILQRITNSRIEIIFNPSADI 886
Clustal Consensus   .. :   . *  :.:*:: ***::  :: :: :: * .:.:.:*

                           1060      1070      1080      1090      1100
                   ....|....|....|....|....|....|....|....|....|....|
HUGT1              SDMPLKSFYRYVLEPEISFTSDNSFAKGPIAKFLDMPQSPLFTLNLNTPE 1078
HUGT2              SEAPLESFYRFVLEPELMSGANDVSSLGPVAKFLDIPESPLLILNMITPE 1056
CeUGGT1            SELPLKRFYRYAAASELSFDHNGNLNTN-VVREDNLPSKQLLTLSLQAPD 1035
CeUGGT2            QEMPIKRFYRFVANEKLLFNEDG-SMENHSVVFSNLPQKQLLTMSLETND 935
Clustal Consensus  .: *:: ***:.   ::    :.    .  . * ::*.. *: :.: : : 330

                           1110      1120      1130      1140      1150
                   ....|....|....|....|....|....|....|....|....|....|
HUGT1              SWMVESVRTPYDLDNIYLEEVDSVVAAEYELEYLLLEGHCYDITTGQPPR 1128
HUGT2              GWLVETVHSNCDLDNIHLKDTEKTVTAEYELEYLLLEGQCFDKVTEQPPR 1106
CeUGGT1            SWIVEAVSAKYDLDNIKMEQANGDVTAEFALQHLLLDGQCFDEVSGQPPR 1085
CeUGGT2            AWMIEVKKAEYDLDNILLETASEDVEAVYSLEHILVEGTSR-KMSGEASD 984
Clustal Consensus  .*::*   :  ***** :: ..  * * : *:::*::* .    : :..  355

                           1160      1170      1180      1190      1200
                   ....|....|....|....|....|....|....|....|....|....|
HUGT1              GLQFTLGTSANPVIVDTIVMANLGYFQLKANPGAWILRLRKGRSEDIYRI 1178
HUGT2              GLQFTLGTKNKPAVVDTIVMAHHGYFQLKANPGAWILRLHQGKSEDIYQI 1156
CeUGGT1            GLQFTLGTDKNPKQFDTIVMANLGYFQLKANPGAWKLEIRDGKSSEIYKI 1135
CeUGGT2            GLEVELSSGGKN--YDTIVMLNLGYFQLKAEPGVWNLHLRNGHSADEHKI 1032
Clustal Consensus  **:. *.:  :    ***** : *******:**.* *.::.*:* : ::* 388

                           1210      1220      1230      1240      1250
                   ....|....|....|....|....|....|....|....|....|....|
HUGT1              YSHDGTDSPPDADEVVIVLNNFKSKIIKVKVQKKADMVNEDLLSDGTSEN 1228
HUGT2              VGHEGTDSQADLEDIIVVLNSFKSKILKVKVKKETDKIKEDILTD-EDEK 1205
CeUGGT1            GSHVGAEKIG-EDVLQVVIDSFTGKSVRVRVEKREGMEERNLLSD----D 1180
CeUGGT2            VTID---SIPVENDIQIVVDSFSGKWVELSVEELTEPKESDDELS----- 1074
Clustal Consensus         .    : : :*::.*..* :.: *::     : :   .

                           1260      1270      1280      1290      1300
                   ....|....|....|....|....|....|....|....|....|....|
HUGT1              ESGFWDSFKWGFTGQKTEEVKQDKDDIINIFSVASGHLYERFLRIMMLSV 1278
HUGT2              TKGLWDSIKS-FTVSLHKENKKEKD-VLNIFSVASGHLYERFLRIMMLSV 1253
CeUGGT1            EEGVWSSLSN------LVSSKEKTQEVINVFSIASGHLYERFMRIMIVSV 1224
CeUGGT2            IESLLNSAKN-------YFASPEPSEVINVFSIASGHLYERFMRIMMTSV 1117
Clustal Consensus   ... .* .           . . . ::*:**:*********:***: **

                           1310      1320      1330      1340      1350
                   ....|....|....|....|....|....|....|....|....|....|
HUGT1              LKNTKTP-VKFWFLKNYLSPTFKEFIPYMANEYNFQYELVQYKWPRWLHQ 1327
HUGT2              LRNTKTP-VKFWLLKNYLSPTFKEVIPHMAKEYGFRYELVQYRWPRWLRQ 1302
CeUGGT1            MKNTKHP-VKFWLLKNYLSPQFKETLPTLAKHYGFEYELIEYKWPRWLHQ 1273
CeUGGT2            LNNTKTQKVKFWLLKNYLSPKFKETIPKLAEFYKFEFELVEYKWPKWLHK 1167
Clustal Consensus  :.***   ****:******* *** :* :*: * *.:**::*:**:**:: 467
```

```
CeUGGT2            LNNTKTQKVKFWLLKNYLSPKFKETIPKLAEFYKFEFELVEYKWPKWLHK 1167
Clustal Consensus  :.***   ****:******* *** :* :*: * *.:**::*:**:**:: 467

                            1360      1370      1380      1390      1400
                   ....|....|....|....|....|....|....|....|....|....|
HUGT1              QTEKQRIIWGYKILFLDVLFPLVVDKFLFVDADQIVRTDLKELRDFNLDG 1377
HUGT2              QTERQRIIWGYKILFLDVLFPLAVDKIIFVDADQIVRHDLKELRDFDLDG 1352
CeUGGT1            QKEKQRIMWGFKILFLDVLFPLDVQKVIFVDADQVVRADLMELMKFDLGN 1323
CeUGGT2            QTEKQRVMWGYKILFLDVLFPLNVDKIIFVDADQVVRADLQELMDFNLNG 1217
Clustal Consensus  *.*:**::**:*********** *:*.:******:** ** ** .*:*.. 508

                            1410      1420      1430      1440      1450
                   ....|....|....|....|....|....|....|....|....|....|
HUGT1              APYGYTPFCDSRREMDGYRFWKSGYWASHLAGRKYHISALYVVDLKKFRK 1427
HUGT2              APYGYTPFCDSRREMDGYRFWKTGYWASHLLRRKYHISALYVVDLKKFRR 1402
CeUGGT1            APYGYVPFCESRKEMDGFRFWKQGYWANHLAGRRYHISALYVIDLQKFRQ 1373
CeUGGT2            APYGYVPFCESRTEMDGFRFWKSGYWKNHLMGRKYHISALYVVDLKAFRE 1267
Clustal Consensus  *****.***:** ****:**** *** .**  *:********:**: **. 549

                            1460      1470      1480      1490      1500
                   ....|....|....|....|....|....|....|....|....|....|
HUGT1              IAAGDRLRGQYQGLSQDPNSLSNLDQDLPNNMIHQVPIKSLPQEWLWCET 1477
HUGT2              IGAGDRLRSQYQALSQDPNSLSNLDQDLPNNMIYQVAIKSLPQDWLWCET 1452
CeUGGT1            IAAGDRLRGQYQGLSGDPNSLANLDQDLPNNMIHQVKIKSLPQEWLWCET 1423
CeUGGT2            FSAGDRLRGRYDSLSADPNSLSNLDQDLPNNMLHEVPIKSLPQEWLWCET 1317
Clustal Consensus  :.******.:*:.** *****:**********:::* ******:****** 594

                            1510      1520      1530      1540      1550
                   ....|....|....|....|....|....|....|....|....|....|
HUGT1              WCDDASKKRAKTIDLCNNPMTKEPKLEAAVRIVPEWQDYDQEIKQLQIRF 1527
HUGT2              WCDDESKQRAKTIDLCNNPKTKESKLKAAARIVPEWVEYDAEIRQLLDHL 1502
CeUGGT1            WCDDGSKKNAKTIDLCNNPLTKEPKLDSAARIIGEWKTYDDEIREVISGH 1473
CeUGGT2            WCDDGSKEKAKTIDLCNNPLTKEPKLNSAKRIIKEWTEYDSEISKVLNSA 1367
Clustal Consensus  **** **:.********** ***.**.:* **: **  ** ** ::     629

                            1560      1570
                   ....|....|....|....|....|...
HUGT1              QKEKETGALYKEKTKEPSREGPQKREEL 1555
HUGT2              ENKKQDTILTHDEL-------------- 1516
CeUGGT1            SSDNPSDNVISENDDSHTEL-------- 1493
CeUGGT2            DINTPSPSVDRDEL-------------- 1381
Clustal Consensus  . ..    :  ::                632
```
